# Ultra-broadband reflectionless Brewster absorber protected by reciprocity

**DOI:** 10.1038/s41377-021-00529-2

**Published:** 2021-04-23

**Authors:** Jie Luo, Hongchen Chu, Ruwen Peng, Mu Wang, Jensen Li, Yun Lai

**Affiliations:** 1 School of Physical Science and Technology, Soochow University, Suzhou, 215006 China; 2 National Laboratory of Solid State Microstructures, School of Physics, and Collaborative Innovation Center of Advanced Microstructures, Nanjing University, Nanjing, 210093 China; 3 Department of Physics, The Hong Kong University of Science and Technology, Clear Water Bay, Hong Kong, China; 4 William Mong Institute of Nano Science and Technology, The Hong Kong University of Science and Technology, Clear Water Bay, Kowloon, Hong Kong, China

**Keywords:** Metamaterials, Sub-wavelength optics

## Abstract

The Brewster’s law predicts zero reflection of p-polarization on a dielectric surface at a particular angle. However, when loss is introduced into the permittivity of the dielectric, the Brewster condition breaks down and reflection unavoidably appears. In this work, we found an exception to this long-standing dilemma by creating a class of nonmagnetic anisotropic metamaterials, where anomalous Brewster effects with independently tunable absorption and refraction emerge. This loss-independent Brewster effect is bestowed by the extra degrees of freedoms introduced by anisotropy and strictly protected by the reciprocity principle. The bandwidth can cover an extremely wide spectrum from dc to optical frequencies. Two examples of reflectionless Brewster absorbers with different Brewster angles are both demonstrated to achieve large absorbance in a wide spectrum via microwave experiments. Our work extends the scope of Brewster effect to the horizon of nonmagnetic absorptive materials, which promises an unprecedented wide bandwidth for reflectionless absorption with high efficiency.

## Introduction

In the early 1810s, Sir David Brewster experimentally showed that when unpolarized light impinges on a dielectric interface, the reflected light would be linearly polarized if the reflected beam is perpendicular to the refracted one^1,2^. The origin of this Brewster effect lies in the elimination of reflection that occurs for the transverse-magnetic (TM)-polarized light at the Brewster’s angle. However, when loss is introduced to the permittivity of dielectrics, a phase difference between the electric and magnetic fields appears, which introduces substantial reflection and breaks the Brewster condition^3,4^. Consequently, for the purpose of reflectionless absorption, complex engineering on the material permeability and dispersions^5,6^ has been generally applied, which, however, introduces tremendous difficulty in the widening of the bandwidth.

Recently, with the rise of metamaterials that go beyond natural materials in many aspects, a lot of efforts have been devoted to the reexamination of the Brewster effect^7–17^. Generalized Brewster effect has been realized for enhanced transmission at selected angles^13,17^. Wide-angle Brewster effect has also been realized^14–16^. The bandwidth is relatively limited for resonant meta-structures^13–16^, but it can be extended to an extremely wide spectrum from dc to THz and even higher frequencies for effective media such as the metallic gratings^8–11^. Despite these inspiring achievements, these generalized Brewster effects still face the same handicap as the traditional Brewster effect: unavoidable reflection at the presence of large absorption.

In this work, by designing anisotropic metamaterials, we reveal an anomalous Brewster effect (ABE) that allows independently tunable absorption and refraction in an ultra-broadband spectrum from dc to optical frequencies. No matter how large the absorption is, zero reflection can be maintained for p-polarization, which is impossible in previous traditional and generalized Brewster effects. Such an amazing effect is bestowed by anisotropic metamaterials designed under the guidance of the reciprocity principle. In this metamaterial, the anisotropy introduces extra degrees of freedoms to tune the absorption and refraction without affecting the Brewster condition. Therefore, loss-independent Brewster effect can be realized where the damping can be sufficiently large to achieve almost total absorption within a thickness of 1–2 wavelengths. By performing proof-of-principle experiments in the microwave regime, we have successfully verified the theory. Our work provides a practical route to solve the long-standing dilemma between the Brewster effect and absorption.

**Fig. 1:**
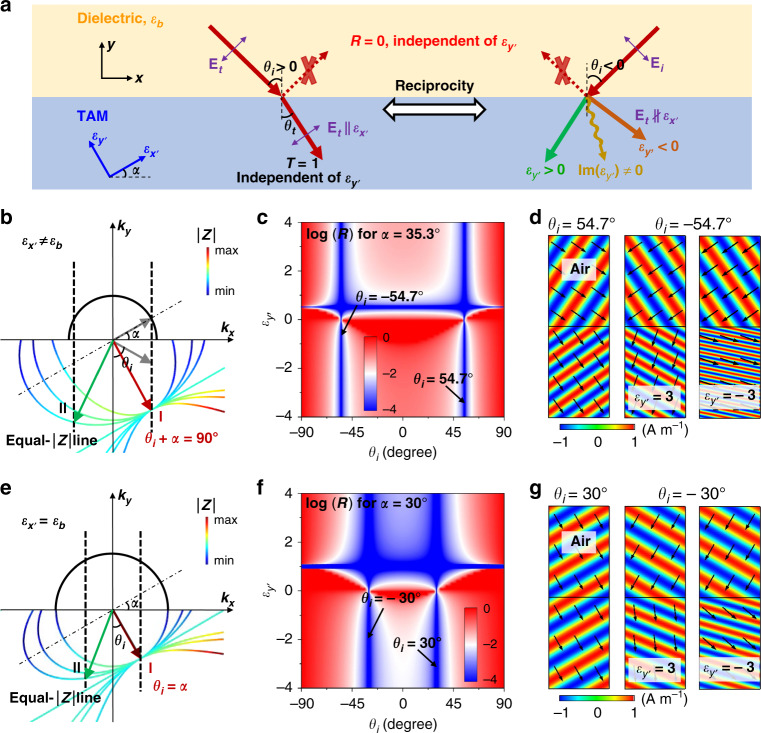
Simultaneous realization of $\varepsilon _{{\boldsymbol{y}}\prime }$-independent Brewster effect and $\varepsilon _{{\boldsymbol{y}}\prime }$-controlled transmission in TAMs. **a** Illustration of the Brewster effect between a background dielectric $\varepsilon _b$ and a nonmagnetic TAM. Due to the protection of reciprocity, the wave impedance in the left case $\theta _i \,>\, 0$ is exactly the same as that in the right case $\theta _i \,<\, 0$. The transmission in the right (left) case is dependent (independent) on $\varepsilon _{y\prime }$. The upper plane of EFCs of the background dielectric (black solid lines), and the lower plane of EFCs of the TAM when $\varepsilon _{x\prime }$ and $\alpha$ are fixed but $\varepsilon _{y\prime }$ is varied (color solid lines), regarding the cases of **b** $\varepsilon _{x\prime } \,\ne\, \varepsilon _b$ and **e** $\varepsilon _{x\prime } = \varepsilon _b$, respectively. The colors denote the magnitude of wave impedance $\left| Z \right|$. The black dashed vertical lines are the equal-$\left| Z \right|$ lines, which intersect with the EFCs at points I and II, respectively. The gray arrows denote the wave vectors of the incident and reflected beams determined by the EFCs of the background dielectric, and the red and green arrows denote the refracted beam determined by the EFCs of the TAM. Calculated reflectance as functions of $\theta _i$ and $\varepsilon _{y\prime }$ for TM-polarized waves incident from free space onto the TAM with **c** $\varepsilon _{x\prime } = 2$ and $\alpha = 35.3^ \circ$, or **f** $\varepsilon _{x\prime } = 1$ and $\alpha = 30^ \circ$, respectively. Simulated distributions of magnetic fields (color) and group velocity (arrows) for the TAM with **d** $\varepsilon _{x\prime } = 2$ and $\alpha = 35.3^ \circ$ under $\theta _i = 54.7^ \circ$ (left) or $\theta _i = - 54.7^ \circ$ (middle and right), or **g** $\varepsilon _{x\prime } = 1$ and $\alpha = 30^ \circ$ under $\theta _i = 30^ \circ$ (left) or $\theta _i = - 30^ \circ$ (middle and right), respectively. We set $\varepsilon _{y\prime } = 3$ (left and middle) or $\varepsilon _{x\prime } = - 3$ (right).

The schematic graph of our idea is shown in Fig. 1a. The anisotropic metamaterial is designed according to the reciprocity principle. The reciprocity principle requires that the response of a propagation channel is symmetric when the source and detection points are interchanged. For a wave incident and reflected on the surface of a reciprocal material^18^, the switch between the incident angles of *θ*_*i*_ and −*θ*_*i*_ is equivalent to the exchange between the source and detection. Therefore, according to the reciprocity principle, the reflection coefficients are exactly the same for incident angles $\pm \theta _i$, i.e., $r\left( { - \theta _i} \right) = r\left( {\theta _i} \right)$^18,19^. As a result, when the Brewster effect emerges, it is always simultaneously satisfied for $\pm \theta _i$. However, interestingly, upon the change between $\theta _i$ and $- \theta _i$, the absorption and transmission can be changed dramatically. And this makes the ABE possible.

The anisotropic metamaterial is designed such that a trivial solution of the Brewster effect (with zero loss and fixed angle of refraction) is obtained at $\theta _i\, >\, 0$ in an ultra-broad spectrum. At the same time, a nontrivial ABE can always be obtained at $\theta _i\, <\, 0$, where the absorption and refraction can be independently tuned by the extra parameters introduced by anisotropy. When the material is lossless, the angle of refraction can be tuned flexibly from positive to negative. When the material is lossy, ultra-broadband and controllable absorption can be achieved. Reflectionless Brewster absorbers can thus be realized by such nonresonant and nonmagnetic metamaterials, whose working bandwidth in principle can cover an extremely wide spectrum from dc to the optical regime. This result is remarkable as the bandwidth goes significantly beyond those of current techniques utilizing complex engineering of the material permeability and dispersions^5,6^, where a tremendous amount of effort has been devoted. This huge bandwidth attributes to the inherent frequency independence of the Brewster effect.

## Results

Without loss of generality, we consider a TM-polarized wave incident on a nonmagnetic tilted anisotropic medium (TAM) under the incident angle of $\theta _i$, as illustrated in Fig. 1a. The background is a dielectric medium with isotropic permittivity $\varepsilon _b$. The optical axis (along with the permittivity component $\varepsilon _{x^{\prime}}$) of the TAM has a tilt angle $\alpha$ with respect to the $x$ axis. For $\theta _i \,>\, 0$, we find that there are two trivial solutions for realizing the Brewster effect between the background and TAM, which is independent of $\varepsilon _{y^{\prime}}$. One solution for $\varepsilon _{x^{\prime}} \,\ne \, \varepsilon _b$ requires a condition of $\theta _i = \pi /2 - \alpha$ ($\alpha = \arctan \left( {\sqrt {\varepsilon _b} /\sqrt {\varepsilon _{x^{\prime}}} } \right)$), which is exactly the condition of the traditional Brewster effect. The other solution for $\varepsilon _{x^{\prime}} = \varepsilon _b$ requires a condition of $\theta _i = \alpha$ ($\alpha$ is arbitrary), which is also easily understandable (see Supplementary Information). Intriguingly, in both cases, the waves incident at $\theta _i \,>\, 0$ cannot “see” $\varepsilon _{y^{\prime}}$ as the electric field of the refracted beam ${\bf{E}}_t$ is parallel to the direction of $\varepsilon _{x^{\prime}}$. This inspires us to utilize the new parameter $\varepsilon _{y^{\prime}}$ as a control parameter without affecting the impedance matching condition. In the following, we provide a more comprehensive picture based on the equal-frequency contours (EFCs) of the TAM.

Figure 1b, e shows the EFCs of the TAM with fixed $\varepsilon _{x^{\prime}}$ and $\alpha$ for different $\varepsilon _{y\prime }$ under the conditions of $\varepsilon _{x^{\prime}}\, \ne\, \varepsilon _b$ and $\varepsilon _{x^{\prime}} = \varepsilon _b$, respectively. It is seen that in both figures, all the EFCs of the TAM cross a fixed point in the $k_y \,<\, 0$ space, which is marked as the point I (at $k_x = \sqrt {\varepsilon _{x^{\prime}}} k_0\sin \alpha$, $k_y = - \sqrt {\varepsilon _{x^{\prime}}} k_0\cos \alpha$, where $k_0$ is the wave number in free space). When the propagating state at the point I is excited in the TAM (by a proper incident angle $\theta _i \,>\, 0$), the electric field of the refracted wave ${\bf{E}}_t$ is polarized along the direction of $\varepsilon _{x^{\prime}}$, indicating that the waves cannot be influenced by $\varepsilon _{y^{\prime}}$. Here, the wave impedance $Z$ is defined as the ratio of $E_x/H_z$, where $E_x$ and $H_z$ are, respectively, the $x$ component of electric fields and the $z$ component of magnetic fields at the interface (normal to $\widehat y$). Through some derivation, we obtain $\left| Z \right| = Z_0\cos \alpha /\sqrt {\varepsilon _{x^{\prime}}}$ at the point I for the TAM, which confirms that $\left| Z \right|$ is independent of $\varepsilon _{y^{\prime}}$. Here, $Z_0$ is the impedance of vacuum (~377 Ω). More importantly, the same impedance $\left| Z \right| = Z_0\cos \alpha /\sqrt {\varepsilon _{x^{\prime}}}$ is invariant along a vertical line of $k_x = \sqrt {\varepsilon _{x^{\prime}}} k_0\sin \alpha$ for various EFCs, which goes through point I, when we vary $\varepsilon _{y^{\prime}}$. This effect can be clearly seen in Fig. 1b, e where $\left| Z \right|$ is plotted in colors in the EFCs. Therefore, this vertical line is denoted as an equal-$\left| Z \right|$ line (black dashed lines) here. The analytical proof is summarized in Supplementary Information.

Here, we would like to emphasize that despite the two trivial solutions of the Brewster effect for $\theta _i\, >\, 0$ are both independent of $\varepsilon _{y^{\prime}}$, they cannot provide the flexible manipulation of refraction or absorption yet as we mentioned in the introduction. This is because refracted waves cannot “see” $\varepsilon _{y^{\prime}}$ and therefore, cannot be influenced by $\varepsilon _{y^{\prime}}$ in any way. However, it becomes interesting when the reciprocity principle is applied. Now, we consider flipping the sign of the incident angle from $\theta _i \,>\,0$ to $\theta _i \,<\, 0$ with the magnitude unchanged. According to the reciprocity principle, the Brewster effect is guaranteed and independent of $\varepsilon _{y^{\prime}}$. Therefore, we have another equal-$\left| Z \right|$ line at $k_x = - \sqrt {\varepsilon _{x^{\prime}}} k_0\sin \alpha$, as marked by black dashed lines in Fig. 1b, e. In the case of $\theta _i\, <\, 0$, the propagating states at point II are excited in the TAM, where the equal-$\left| Z \right|$ line and EFC intersect. Obviously, the EFC and the refraction angle both vary significantly with $\varepsilon _{y^{\prime}}$. In this situation, the electric field of the refracted wave ${\bf{E}}_t$ is no long parallel to the direction of $\varepsilon _{x^{\prime}}$, bestowing tunable refractive behaviors by changing $\varepsilon _{y^{\prime}}$, while no reflection is guaranteed by reciprocity at the same time. When $\varepsilon _{y^{\prime}}$ has an imaginary part, the absorption can be introduced. Therefore, we have obtained two nontrivial solutions of the ABE with tunable refraction and absorption.

In the following, we perform numerical simulations to prove the reciprocity-protected ABE with tunable refraction in the background of air (i.e., $\varepsilon _b = 1$). The simulations were performed by the commercial finite-element-method software COMSOL Multiphysics. Here, we consider TM-polarized waves incident on the TAM. First, the TAM is chosen to be $\varepsilon _{x^{\prime}} = 2$ and $\alpha = \arctan \left( {\sqrt {\varepsilon _b} /\sqrt {\varepsilon _{x^{\prime}}} } \right) = 35.3^ \circ$. The reflectance on the air-TAM interface as a function of the $\theta _i$ and $\varepsilon _{y^{\prime}}$ is plotted in Fig. 1c. Clearly, near-zero reflection is observed around $\theta _i = \pm 54.7^ \circ {\mathrm{ = }} \pm \left( {90^ \circ - \alpha } \right)$, irrespective of $\varepsilon _{y^{\prime}}$. We also notice horizontal blue lines related to omnidirectional Brewster effect, which can be understood through coordinate transformation^16,20^. In Fig. 1d, we plot the distributions of magnetic fields (color) and group velocity (arrows) for the cases of $\theta _i = 54.7^ \circ$ (left), $\theta _i = - 54.7^ \circ$ and $\varepsilon _{y^{\prime}} = 3$ (middle), and $\theta _i = - 54.7^ \circ$ and $\varepsilon _{y^{\prime}} = - 3$ (right), respectively. In all three cases, the reflection clearly disappears. By tuning $\varepsilon _{y^{\prime}}$ from 3 to −3, the angle of refraction is evidently changed from positive to negative, which confirms the controllability of refraction by tuning $\varepsilon _{y^{\prime}}$. Second, we consider a TAM with $\varepsilon _{x^{\prime}} = 1$. In this situation, $\alpha$ can be arbitrary. Here, we take $\alpha = 30^ \circ$ as an example. The reflectance on the air-TAM interface is plotted in Fig. 1f, which clearly shows $\varepsilon _{y^{\prime}}$-independent near-zero reflection under $\theta _i = \pm 30^ \circ$, i.e., $\theta _i = \pm \alpha$. The reflectionless refraction for $\theta _i = \pm 30^ \circ$, as well as the controllable refraction for $\theta _i = - 30^ \circ$ have also been well confirmed by simulations, as shown in Fig. 1g.

It is worth noting that when $\varepsilon _{y^{\prime}}$ approaches infinity, the EFCs of the TAM turn into two parallel lines with constant wave impedance $\left| Z \right| = Z_0\cos \alpha /\sqrt {\varepsilon _{x^{\prime}}}$, as shown as cyan color in Fig. 1b, e. Such a case can be realized by using a tilted aluminum film array, which leads to ultra-broadband reflectionless negative refraction (see Supplementary Information).

As an important consequence of the above reciprocity-protected ABE, we can further let $\varepsilon _{y^{\prime}}$ be a complex number and introduce loss into the system. Such reciprocity consideration allows us to realize perfect-impedance-matched absorbing materials with unprecedented wide bandwidth, which is extremely difficult via other approaches, if not impossible. Such absorbers are denoted as reflectionless Brewster absorbers here. In the following, we demonstrate the design and realization of this ultra-broadband reflectionless Brewster absorbers by using tilted conductive film (CF) arrays.

**Fig. 2:**
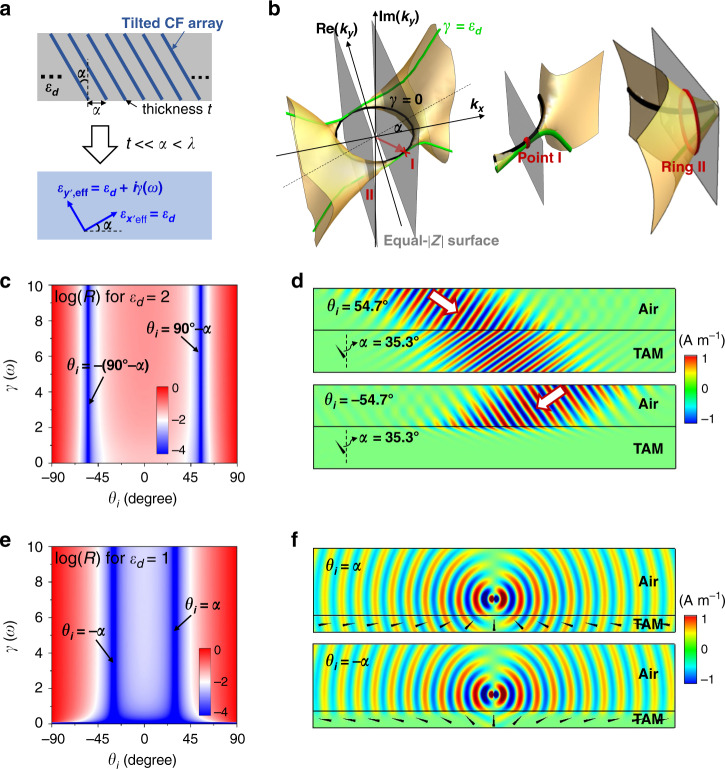
Ultra-broadband Brewster effect with controllable absorption by using tilted CF arrays. **a** Illustrations of a tilted CF array embedded in a dielectric of $\varepsilon _d$ (upper) as the effective TAM (lower). **b** The equal-frequency surface of the effective TAM that is formed by varying $\gamma \left( \omega \right)$. The black and green lines denote the EFCs regarding the cases of $\gamma \left( \omega \right) = 0$ and $\gamma \left( \omega \right) = \varepsilon _d$, respectively. The gray surfaces denote the equal-$\left| Z \right|$ surfaces, which intersect with the equal-frequency surface at point I and the ring II (enlarge insets in the right). Calculated reflectance as functions of the $\theta _i$ and $\gamma \left( \omega \right)$ for TM-polarized waves incident onto the effective TAM with **c** $\varepsilon _d = 2$ and $\alpha = 35.3^ \circ$, or **e** $\varepsilon _d = 1$ and $\alpha = 30^ \circ$, respectively. **d** Simulated magnetic-field distributions when a TM-polarized Gaussian beam is incident onto the effective TAM with $\varepsilon _d = 2$, $\alpha = 35.3^ \circ$ and $\gamma \left( \omega \right) = 3$ for $\theta _i = 54.7^ \circ$ (upper) or $\theta _i = - 54.7^ \circ$ (lower). **f** Simulated magnetic-field distributions for a TM-polarized dipole source above an inhomogeneous TAM with fixed $\varepsilon _d = 1$ and $\gamma \left( \omega \right) = 3$ but varied $\alpha$. The $\alpha$ is adjusted along the surface so that the condition of $\theta _i = \alpha$ (upper) or $\theta _i = - \alpha$ (lower) is satisfied everywhere. The arrows in (**d**, **f**) denote the orientation of the axis $\varepsilon _{y\prime }$.

The tilted CF array is embedded in a dielectric host of $\varepsilon _d$ under a tilt angle of $\alpha$, as illustrated by the upper panel graph of Fig. 2a. The separation distance between two adjacent CFs is $a$, which is much larger than the thickness of the CFs $t$ (i.e., $a \, > > \, t$), but smaller than the wavelength $\lambda$ in the dielectric host (i.e., $a \,<\, \lambda$). Under such circumstances, the tilted CF array can be approximately homogenized as an effective TAM (the lower panel) with $\varepsilon _{x^{\prime} ,{\rm{eff}}} = \varepsilon _d$ and $\varepsilon _{y^{\prime} ,{\rm{eff}}} = \varepsilon _d + i\gamma \left( \omega \right)$, where $\gamma \left( \omega \right) = \left( {Z_0/R_s} \right)/\,\left( {k_0a\cos \alpha } \right)$ is a function of angular frequency $\omega$. Here, $R_s$ is the sheet resistance of the CFs. Figure 2b presents the equal-frequency surface of the effective TAM in the three-dimensional *k* space composed of the $k_x$, ${\rm{Re}} \left( {k_y} \right)$, and ${\rm{Im}} \left( {k_y} \right)$ coordinates, which is obtained by adopting different values of $\gamma \left( \omega \right)$. The EFCs regarding the particular cases $\gamma \left( \omega \right) = 0$ and $\gamma \left( \omega \right) = \varepsilon _d$ are plotted as black and green lines, respectively. In the absence of material loss ($\gamma \left( \omega \right) = 0$), the EFC is a circle on the $k_x{\mathrm{ - }}{\rm{Re}} \left( {k_y} \right)$ plane. When loss is introduced, the EFC separates into two curves extending into the ${\rm{Im}} \left( {k_y} \right)$ direction. Interestingly, we find that the EFCs of the TAM with any $\gamma \left( \omega \right)$ always pass through a fixed point I located at the coordinates $k_x = \sqrt {\varepsilon _d} k_0\sin \alpha$, ${\rm{Re}} \left( {k_y} \right) = - \sqrt {\varepsilon _d} k_0\cos \alpha$, and ${\rm{Im}} \left( {k_y} \right) = 0$ (see the enlarged inset graph), indicating that it is independent of $\gamma \left( \omega \right)$. Similar to the lossless case, we have an equal-$\left| Z \right|$ surface at $k_x = \sqrt {\varepsilon _d} k_0\sin \alpha$, which crosses the point I, and another equal-$\left| Z \right|$ surface at $k_x = - \sqrt {\varepsilon _d} k_0\sin \alpha$, whose intersection with the equal-frequency surface turns into the ring II, as shown in Fig. 2b. At the point I and on the ring II, the wave impedance is always a constant $\left| Z \right| = Z_0\cos \alpha /\sqrt {\varepsilon _d}$. Interestingly, on the ring II, the induced ${\rm{Im}} \left( {k_y} \right) \,\ne \,0$ by $\gamma \left( \omega \right) \,\ne\, 0$ indicates the intriguing possibility to achieve large absorption without breaking the impedance matching condition.

We have also performed numerical simulations to verify the reciprocity-protected ABE with large absorption. Here, we consider TM-polarized waves incident on the TAM from an air background. First, the TAM is set as $\varepsilon _d = 2$ and $\alpha = 35.3^ \circ$. In Fig. 2c, we plot the reflectance on the air-TAM interface as a function of $\theta _i$ and $\gamma \left( \omega \right)$, which clearly shows a regime of near-zero reflection around $\theta _i = \pm 54.7^ \circ$, irrespective of $\gamma \left( \omega \right)$. In Fig. 2d, we plot the simulated magnetic-field distributions for Gaussian beams incident on a TAM with $\gamma \left( \omega \right) = 3$ at $\theta _i = \pm 54.7^ \circ$. Clearly, for $\theta _i = 54.7^ \circ$ (the upper panel), the Gaussian beam transmits through the TAM, with almost no reflection or absorption. While for $\theta _i = - 54.7^ \circ$ (the lower panel), the Gaussian beam is almost totally absorbed by the TAM, with no reflection or transmission. Second, the TAM is set as $\varepsilon _d = 1$. The reflectance on the air-TAM interface is plotted in Fig. 2e, in which the TAM has $\alpha = 30^ \circ$. Clearly, near-zero reflection emerges around $\theta _i = \pm 30^ \circ$, irrespective of $\gamma \left( \omega \right)$. Interestingly, in this case, $\alpha$ is arbitrary and the Brewster effect is realized as long as $\theta _i = \pm \alpha$. This characteristic could be valuable for the absorption of the near field. For a demonstration, in Fig. 2f, we demonstrate the simulated magnetic-field distributions when a dipole source is placed above two inhomogeneous TAMs with variant $\alpha$ along the surface. The condition of $\theta _i = \alpha$ (the upper panel) or $\theta _i = - \alpha$ (the lower panel) is satisfied everywhere on the surface of the TAMs, where the $\gamma \left( \omega \right)$ is set as 2 and the orientation of the axis $\varepsilon _{y\prime }$ is denoted by arrows. Interestingly, in the case of $\theta _i = \alpha$, the radiation waves of the dipole source are almost totally transmitted through the TAM, with no reflection. While in the case of $\theta _i = - \alpha$, almost all the radiated waves from the dipole source are absorbed, with no reflection. This indicates a transition from total transparency to total absorption, simply by varying the orientation of the axis of the TAM. In other words, we first insert the parallel plates in a way that they do not perturb the fields as all these parallel plates act as waveguides without cut-off to allow unit transmittance. Then, all these conducting plates have the orientation flipped horizontally, allowing reciprocity-protected zero reflection, while locally the E-fields have components parallel to the plates to generate huge absorption.

### Experimental observation of ultra-broadband reflectionless Brewster absorbers

**Fig. 3:**
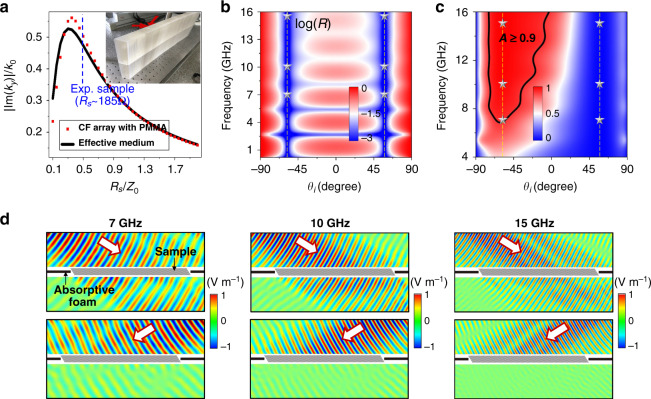
Experimental observation of ultra-broadband reflectionless Brewster absorbers in the case of *ε*_*d ≠*_*ε*_*b*_. **a** The $\left| {{\rm{Im}} \left( {k_y} \right)} \right|$ in the CF array (red dots) and the corresponding effective medium (black solid lines) as the function of the $R_s$ at 10 GHz. The inset shows the picture of the fabricated sample consisting of alternative PMMA and ITO films $\alpha = 31.8^ \circ$. The thickness and height of the sample are 30 and 200 mm, respectively. Simulated **b** reflectance and **c** absorbance on the fabricated sample as functions of the $\theta _i$ and working frequency, respectively. The stars denote the measured cases in experiments. **d** Measured electric field distributions under $\theta _i = 58.2^ \circ$ (upper) and $\theta _i = - 58.2^ \circ$ (lower) at 7 GHz (left), 10 GHz (middle), and 15 GHz (right). The black thick lines near the sample denote the absorptive foam.

Since the reciprocity-protected ABE is irrespective of $\gamma \left( \omega \right)$, ultra-broadband reflectionless absorbers of electromagnetic waves can be realized. In the following, by using the tilted CF arrays, we experimentally verify this phenomenon in microwave frequencies. A sample of the CF array is fabricated, which consists of alternative polymethyl methacrylate (PMMA, relative permittivity ~2.6) and indium tin oxide (ITO) films (thickness ~100 μm, as CFs) with a separation distance of $a$ = 5 mm. The sample has a tilt angle of $\alpha = 31.8^ \circ$, a thickness of 30 mm, and a height of 200 mm, as shown by the photo shown in Fig. 3a. Ultra-broadband Brewster effect appears at $\theta _i = \pm 58.2^ \circ$ for such a TAM, while absorption only emerges at $\theta _i = - 58.2^ \circ$. In Fig. 3a, we plot the $\left| {{\rm{Im}} \left( {k_y} \right)} \right|$ in the CF array (red dots) and the corresponding effective medium (black lines) as the function of the sheet resistance $R_s$ of the CFs at 10 GHz. Here, $\left| {{\rm{Im}} \left( {k_y} \right)} \right|$ determines the attenuation rate, and a larger $\left| {{\rm{Im}} \left( {k_y} \right)} \right|$ implies a thinner thickness requirement for the absorber. In Fig. 3a, the maximal $\left| {{\rm{Im}} \left( {k_y} \right)} \right|$ is found to be around the optimal sheet resistance **~**$0.35Z_0$ **=** 132 Ω for the CF array, which coincides well with the effective medium calculation, indicating the validity of effective medium here. In the experiments, each piece of ITO film has a sheet resistance of ~370 Ω. Therefore, we stacked two ITO films into one, so that the composite film has a sheet resistance of ~185 Ω (blue dashed lines in Fig. 3a), and relatively good performance of absorption can be attained. Figure 3b, c shows, respectively, the simulated reflectance and absorbance of the sample. In Fig. 3b, near-zero reflection emerges around $\theta _i = \pm 58.2^ \circ$ for all frequencies below 16 GHz. Above 16 GHz, the non-reflection effect gradually deteriorates because the effective medium turns inaccurate in the short wavelength regime. Nevertheless, we note that the upper working frequency can be significantly raised, even to the optical frequency, by simply decreasing the scale of the structures, i.e., the separation distance. While the absorbance in Fig. 3c shows a distinct asymmetric behavior. Zero absorbance occurs around $\theta _i = 58.2^ \circ$ and high absorbance (>0.9) occurs around $\theta _i = - 58.2^ \circ$ in the frequency regime from 7 to 16 GHz. When the incident angle deviates away from the optimal angle $- 58.2^ \circ$, the absorption is still quite high, showing some angular insensitivity in the absorption performance. Here, we note that the relatively low absorbance below 7 GHz is mainly induced by transmission since the reflectance is almost 0. Since there is no reflection, and waves in the TAM decay exponentially with the propagation distance, the absorption at low frequencies can be easily increased through increasing the sample thickness.

The experimental results are shown in Fig. 3d. In the experiment, an emitting horn antenna is placed ~0.8 meters away from the sample to generate the incident waves. We measured the near-field electric fields before and after the sample by using a probing antenna. The scanning areas are 700 × 150 mm^2^ each, located on the central plane of the sample. In the following, we demonstrate the measured electric field distribution at 7, 10, and 15 GHz (marked by stars in Fig. 3b, c). The upper (lower) panel graph of Fig. 3d presents the measured electric fields at $\theta _i = 58.2^ \circ$ ($\theta _i = - 58.2^ \circ$). Clearly, there is no reflection in all cases, demonstrating the ultra-broadband ABE with absorption. Interestingly, near-total transmission is observed at $\theta _i = 58.2^ \circ$, while large absorption is clearly observed at $\theta _i = - 58.2^ \circ$, for all three selected frequencies. In experiments, we evaluate the absorbance through integrating far-field power for all directions, finding absorbance as high as 0.95, 0.98, and 0.99 at 7, 10, and 15 GHz, respectively. The absorbance at other frequencies in the measuring frequency range 7–15 GHz is also quite high (see Supplementary Information).

**Fig. 4:**
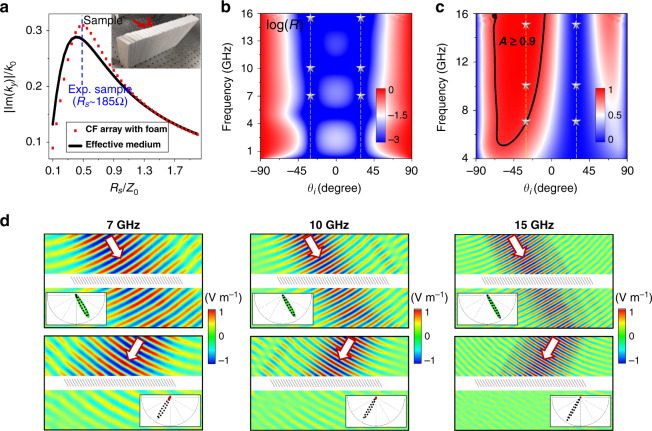
Experimental observation of ultra-broadband reflectionless Brewster absorbers in the case of *ε*_*d*_ = *ε*_*b*_. **a** The $\left| {{\rm{Im}} \left( {k_y} \right)} \right|$ in the CF array (red dots) and the corresponding effective medium (black solid lines) as the function of the $R_s$ at 10 GHz. The inset shows the picture of the fabricated sample consisting of an alternative foam and ITO films. **b**, **c** Simulated reflectance and absorbance on the fabricated sample as functions of the $\theta _i$ and working frequency, respectively. The stars denote the measured cases in experiments. **d** Measured electric field distributions under $\theta _i = 30^ \circ$ (upper) and $\theta _i = - 30^ \circ$ (lower) at 7 GHz (left), 10 GHz (middle), and 15 GHz (right). The insets show the measured far-field radiation patterns with (green and red solid lines) or without (black dashed lines) samples.

In the second experiment, we verify the other nontrivial solution of the ultra-broadband ABE with absorption for the TAM designed with $\varepsilon _d = 1$. In this case, $\alpha$ is arbitrary and the Brewster effect is realized as long as $\theta _i = \pm \alpha$. The TAM sample is composed of alternative foam (relative permittivity ~1) and ITO films (thickness ~100 μm, as CFs) with a separation distance of $a$ = 10 mm. The sample has a tilt angle of $\alpha = 30^ \circ$, a thickness of 30 mm, and a height of 200 mm, as shown by the photo in Fig. 4a. In Fig. 4a, we also plot the $\left| {{\rm{Im}} \left( {k_y} \right)} \right|$ in the sample (red dots) and the corresponding effective medium (black lines) as the function of the sheet resistance $R_s$ of the CFs at 10 GHz. The maximal $\left| {{\rm{Im}} \left( {k_y} \right)} \right|$ is observed around the optimal sheet resistance $Z_0\cos \alpha /\left( {k_0a} \right)$ = 155 Ω. Theoretically, the maximal value $\left| {{\rm{Im}} \left( {k_y} \right)} \right|$ is derived to be $k_0\sin \alpha \tan \alpha$. In the experiment, we have exploited a composite film with $R_s$ ~ 185 Ω consisting of two ITO films as adopted in the previous experiment (see blue dashed lines in Fig. 4a). Figure 4b, c shows, respectively, the simulated reflectance and absorbance of the sample. Clearly, ultra-low reflection is achieved around $\theta _i = \pm 30^ \circ$ for all frequencies below 16 GHz. Near-perfect absorption is achieved around $\theta _i = - 30^ \circ$ in the frequency regime from 7 to 15 GHz, while there is almost no absorption around $\theta _i = 30^ \circ$. Even when the incident angle deviates away from $- 30^ \circ$, the absorption is still quite high. These results were also confirmed by experimental measurement. The measured electric field distributions are plotted in Fig. 4d for $\theta _i = 30^ \circ$ (the upper panel) and $\theta _i = - 30^ \circ$ (the lower panel), respectively, at 7, 10, and 15 GHz. Indeed, near-total transmission is observed at $\theta _i = 30^ \circ$, while large absorption is clearly observed at $\theta _i = - 30^ \circ$, for all three selected frequencies. The inset graphs of Fig. 4d show the measured far-field radiation patterns (green and red lines) of the sample. The black dashed lines denote the reference patterns in the absence of the sample. Clearly, ultra-broadband ABE with near-perfect absorption (>0.87 at 7 GHz, >0.95 at 10 GHz, >0.99 at 15 GHz) is obtained.

There are several ways to further improve the absorption performance of the reflectionless Brewster absorbers demonstrated above. The simplest method is to increase the thickness of the samples since the metamaterial absorber is perfectly impedance-matched to free space. By adding a reflector behind the CF arrays, the angular-asymmetric performance of absorption would be changed into angular-symmetric large absorption, which may be important in practical applications (see Supplementary Information). In the case of $\varepsilon _d = \varepsilon _b$, the rotation angle $\alpha$ can be arbitrary. Interestingly, the maximal value $\left| {{\rm{Im}} \left( {k_y} \right)} \right|$ is derived as $\left| {{\rm{Im}} \left( {k_y} \right)} \right| = k_0\sin \alpha \tan \alpha$, which tends to infinity as $\alpha$ goes to $90^ \circ$. This means that ultra-broadband perfect absorption can be realized even when $\alpha \to 90^ \circ$. Experimentally, we have also fabricated a sample with $\varepsilon _d = 1$ and $\alpha = 45^ \circ$, showing higher absorption (see Supplementary Information). Ultrathin perfect absorbers with extremely thin thickness can also be obtained based on the TAM with hyperbolic dispersions (see Supplementary Information).

## Discussion

**Fig. 5:**
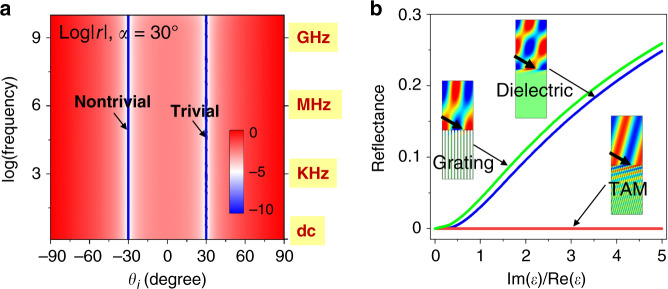
The ultra-broad bandwidth of ABE and the dependence of reflection on absorption. **a** Reflection coefficient as the function of the $\theta _i$ at the interface of free space and the effective medium model of the CF array in Fig. 4. The nontrivial solution corresponds to the ABE that enables flexible large absorption and tunable angle of refraction, while the trivial solution is similar to the traditional Brewster effect, without absorption and tunable refraction. **b** Reflectance as the function of material loss at the interfaces of air-dielectric (blue lines), air-PEC-grating (green lines), and air-TAM (red lines) under $\theta _i = 75^ \circ$. The TAM is composed of subwavelength tilted CF array in a dielectric host with $\varepsilon _d = \tan ^275^ \circ$ and $\alpha = - 15^ \circ$. ${\rm{Re}} \left( \varepsilon \right)$ and ${\rm{Im}} \left( \varepsilon \right)$ denote the real and imaginary parts of the permittivity of the dielectric, or the filling material in grating slits, or the CF of the TAM, respectively. The insets show the simulated magnetic-field distributions when ${\rm{Im}} \left( \varepsilon \right)/{\rm{Re}} \left( \varepsilon \right) = 5$ for the dielectric and grating models, ${\rm{Im}} \left( \varepsilon \right)/{\rm{Re}} \left( \varepsilon \right) = 500$ for the TAM model.

The hallmark advantage of the reflectionless Brewster absorbers is that they are based on inherently nonresonant metamaterials, and thus the bandwidth of impedance-matched absorption can cover the ultra-broad spectrum from dc to optical frequencies, which is far beyond those of other absorber techniques^5,6,21–36^. As a demonstration, in Fig. 5a, we plot the reflection coefficient at the interface of free space and effective medium of the reflectionless Brewster absorber studied in Fig. 4 as the function of the incident angle, showing that zero reflection at $\theta _i = 30^ \circ$ (trivial case without absorption) and $\theta _i = - 30^ \circ$ (nontrivial case with large absorption by the reflectionless Brewster absorber) from dc to GHz frequencies. There is no lower limit for the working frequency, while the upper limit is determined by the validity of the effective medium approximation. Through the reduction of the microstructure unit (e.g., the thickness and the separation distance of the CFs), the working frequency range of perfect-impedance-matched absorption can, in principle, be further widened to cover an unprecedented broad regime from dc to THz, infrared and even optical regimes (see Supplementary Information). Another fundamental advantage of the reflectionless Brewster absorbers lies in the tunability of the magnitude of material loss as well as the refractive behaviors, which offers significant flexibility.

Finally, we would like to emphasize the difference between the traditional Brewster effect, the perfect electric conductor (PEC) grating structures^8–11^ and the ABE proposed here. It has been observed that ultra-broadband Brewster effect can occur in dispersion-less effective media as well as PEC grating structures. However, when material loss is introduced, such Brewster effects will gradually break down, just like the traditional Brewster effect. The reflection can be dramatic when the loss is large. For verification, we consider a dielectric medium and a PEC grating structure exhibiting a Brewster angle at $75^ \circ$ in the absence of material loss for TM-polarized waves incident from free space. The period $d$ of the grating and the slit width $w$ satisfies $w/d = {\rm{cos}}75^ \circ$. When we gradually increase the loss, dramatic reflection occurs at the air-dielectric and air-grating interfaces, as shown by the simulation results shown in Fig. 5b. Here, ${\rm{Re}} \left( \varepsilon \right)$ and ${\rm{Im}} \left( \varepsilon \right)$ denote, respectively, the real and imaginary parts of the permittivity of the dielectric (${\rm{Re}} \left( \varepsilon \right) = \tan ^275^ \circ$ to ensure a Brewster angle at $75^ \circ$), or the filling material in grating slits (${\rm{Re}} \left( \varepsilon \right) = 1$). The inset graphs show the simulated magnetic-field distribution when ${\rm{Im}} \left( \varepsilon \right)/{\rm{Re}} \left( \varepsilon \right) = 5$. In contrary, when the material loss is introduced to a TAM consisting of subwavelength tilted CF arrays in a dielectric host ($\varepsilon _d = \tan ^275^ \circ$, $\alpha = - 15^ \circ$, relative permittivity of CFs $\varepsilon = {\rm{Re}} \left( \varepsilon \right) + i{\rm{Im}} \left( \varepsilon \right)$), the reflection always remains almost zero irrespective of the magnitude of the loss, as shown in Fig. 5b. Even when ${\rm{Im}} \left( \varepsilon \right)/{\rm{Re}} \left( \varepsilon \right) = 500$, the reflection is still negligible, as seen from the magnetic-field distribution in the inset in Fig. 5b.

In conclusion, in this work, we reveal an ABE for ultra-broadband reflectionless manipulation of waves, including tunable absorption and refraction. The ABE, as protected by the reciprocity principle, guarantees zero reflection for one polarization at a particular incident angle. At the same time, the refraction and absorption are flexibly tunable via the extra degrees of freedoms introduced by the anisotropy of metamaterials. The ABE bestows reflectionless Brewster absorbers with an unprecedented wide bandwidth of impedance-matched absorption. While conventional wisdom tells us that addition of loss will destroy the Brewster effect, we have demonstrated that the mechanism of reciprocity protection escapes from such a deficiency. This principle of ABE is universal for any reciprocal materials and for general classical waves.

## Materials and methods

### Simulations

Numerical simulations in this work are performed using the commercial finite-element simulation software COMSOL Multiphysics. In order to simulate the TM-polarized plane wave under oblique incidence in Fig. 1d, g, the left and right boundaries are set as Floquet periodic boundaries, the top boundary is set as port to excite the incident wave. In Fig. 2d, the Gaussian beam is excited by the top port boundary. In Fig. 2f, the TAM is illuminated by an electric dipole source perpendicular to the TAM surface. The distance of the TAM surface equals the wavelength in air. In Fig. 2d, f, perfect matched layers are set in the bottom to absorb the transmission waves in the TAM.

### Experiments

In near-field experiments, an emitting horn antenna is placed ~0.8 meter away from the sample to generate the incident waves. A probing antenna is used to probe the near-field electric fields on the central plane before and after the sample. The scanning rectangular area is of 700 × 150 mm^2^ (600 × 150 mm^2^) before and after the sample in Fig. 3 (Fig. 4). The probing antenna is mounted to a computer controlled translational stage. Both the probing antenna and the emitting horn antenna are connected to a network analyzer (KEYSIGHT PNA Network Analyzer N5224B) for data acquisition. Because of the selectivity of the probing antenna in measurement, only the electric fields perpendicular to the propagation direction are measured. Therefore, in the scan area before the sample, the measured electric fields come from the incident waves and a part of the possible reflection waves. In order to further confirm the zero reflection and near-perfect absorption in experiments, we have also measured the electric fields in the absence of samples and the far-field power radiation patterns.

In far-field experiments, an emitting horn antenna is placed 1 m away from the sample to generate the incident waves. A receiving horn antenna placed at the same distance is used to measure the radiation pattern. The receiving horn antenna can be freely moved around the sample so that we could receive scattering signals in all directions. Both the emitting and receiving horn antennas are connected to a vector network analyzer (KEYSIGHT PNA Network Analyzer N5224B) for data acquisition. The power radiation pattern in the absence of sample is measured as a reference.

## Supplementary information

Supplementary Information for Ultra-broadband reflectionless Brewster absorber protected by reciprocity
