# Supplementary material for: Ultra-broadband reflectionless Brewster absorber protected by reciprocity
Source: Light Sci Appl. 2021 Apr 23;10:89. doi: 10.1038/s41377-021-00529-2 (PMC8062598; doi:10.1038/s41377-021-00529-2)
Supplement: Supplementary file 1 — Supplementary Information for Ultra-broadband reflectionless Brewster absorber protected by reciprocity [file 41377_2021_529_MOESM1_ESM.docx]

**Supplementary Information for**

**Ultra-broadband reflectionless Brewster absorber protected by reciprocity**

Jie Luo^1*#^, Hongchen Chu^2#^, Ruwen Peng^2^, Mu Wang^2^, Jensen Li^3,4*^, Yun Lai^2*^

^1^School of Physical Science and Technology, Soochow University, Suzhou 215006, China

^2^National Laboratory of Solid State Microstructures, School of Physics, and Collaborative Innovation Center of Advanced Microstructures, Nanjing University, Nanjing 210093, China

^3^Department of Physics, The Hong Kong University of Science and Technology, Clear Water Bay, Hong Kong, China

^4^William Mong Institute of Nano Science and Technology, the Hong Kong University of Science and Technology, Clear Water Bay, Kowloon, Hong Kong, China

*Corresponding authors: Yun Lai ([laiyun@nju.edu.cn](mailto:laiyun@nju.edu.cn)); Jie Luo ([luojie@suda.edu.cn](mailto:luojie@suda.edu.cn)); Jensen Li ([jensenli@ust.hk](mailto:jensenli@ust.hk))

#These authors contributed equally: Jie Luo, Hongchen Chu

[1. Derivations of wave impedance and conditions of ABE](#_Toc65318677)

[2. Complementary discussions of the lossless TAM](#_Toc65318678)

[3. Ultra-broadband reflection-less positive and negative refraction, and experimental verification](#_Toc65318679)

[4. Loss-induced breakdown of the Brewster effect in isotropic dielectrics](#_Toc65318680)

[5. ABE and perfect absorption in general TAM with material loss](#_Toc65318681)

[6. Extraordinary ultrathin perfect absorbers by TAM with hyperbolic dispersions](#_Toc65318682)

[7. Effective medium model of tilted CF array and ABE from dc to the GHz regime](#_Toc65318683)

[8. Optimal sheet resistance analysis, details of experimental samples and further experimental results](#_Toc65318684)

[9. Experimental setup and measurement methods](#_Toc65318685)

[10. Improvement of absorption by using reflectors](#_Toc65318686)

## Derivations of wave impedance and conditions of ABE

We consider a transverse-magnetic (TM, magnetic field along the $z$ direction) polarized wave propagating on the $x\text{-}y$ plane. The wave is incident from an isotropic dielectric (relative permittivity $\varepsilon_{b}$) onto a nonmagnetic tilted anisotropic medium (TAM). Such a TAM is characterized by uniaxial relative permittivity components $\varepsilon_{x'}$ and $\varepsilon_{y'}$, together with a rotation angle $\alpha$ (see Fig. 1 in the Main Text). The reflection coefficient (with respect to the magnetic field) at the dielectric-TAM interface can be derived as,

$r^{TM}=\frac{\left(\overline{\varepsilon}_{xx}\overline{\varepsilon}_{yy}-\overline{\varepsilon}_{xy}\overline{\varepsilon}_{yx}\right)k_{b,y}-\varepsilon_{b}\left(\overline{\varepsilon}_{xy}k_{x}+\overline{\varepsilon}_{yy}k_{y}\right)}{\left(\overline{\varepsilon}_{xx}\overline{\varepsilon}_{yy}-\overline{\varepsilon}_{xy}\overline{\varepsilon}_{yx}\right)k_{b,y}+\varepsilon_{b}\left(\overline{\varepsilon}_{xy}k_{x}+\overline{\varepsilon}_{yy}k_{y}\right)}$ (S1)

where $\overline{\varepsilon}_{xy}$ and $\overline{\varepsilon}_{yy}$ are the components of the relative permittivity tensor $\bar{\varepsilon}$ in the un-rotated $x\text{-}y$ coordinate, i.e. $\bar{\varepsilon}=\left(\begin{matrix} \overline{\varepsilon}_{xx} & \overline{\varepsilon}_{xy} \\ \overline{\varepsilon}_{yx} & \overline{\varepsilon}_{yy} \end{matrix}\right)=\left(\begin{matrix} \varepsilon_{x'}\cos^{2}\alpha+\varepsilon_{y'}\sin^{2}\alpha& \left(\varepsilon_{x'}-\varepsilon_{y'}\right)\sin\alpha\cos\alpha\\ \left(\varepsilon_{x'}-\varepsilon_{y'}\right)\sin\alpha\cos\alpha& \varepsilon_{x'}\sin^{2}\alpha+\varepsilon_{y'}\cos^{2}\alpha\end{matrix}\right)$.

The $k_{b,y}$ and $k_{x}$ are connected by the dispersion of the isotropic dielectric, i.e., $k_{x}^{2}+k_{b,y}^{2}=\varepsilon_{b}k_{0}^{2}$ with $k_{0}$ being the wave number in free space. For a certain incident angle $\theta_{i}$, we have $k_{x}=\sqrt{\varepsilon_{b}}k_{0}\sin\theta_{i}$ and $k_{b,y}^{f}=-\sqrt{\varepsilon_{b}}k_{0}\cos\theta_{i}$ for forward propagating waves (or $k_{b,y}^{b}=\sqrt{\varepsilon_{b}}k_{0}\cos\theta_{i}$ for backward propagating waves). The $k_{y}$ and $k_{x}$ are connected by the dispersion of the TAM, i.e. $k_{x}^{2}\overline{\varepsilon}_{xx}+k_{y}^{2}\overline{\varepsilon}_{yy}+k_{x}k_{y}\left(\overline{\varepsilon}_{xy}+\overline{\varepsilon}_{yx}\right)=\left(\overline{\varepsilon}_{xx}\overline{\varepsilon}_{yy}-\overline{\varepsilon}_{xy}\overline{\varepsilon}_{yx}\right)k_{0}^{2}$ or $\left(k_{x}^{2}\varepsilon_{x\text{'}}+k_{y}^{2}\varepsilon_{y\text{'}}\right)\cos^{2}\alpha+\left(k_{y}^{2}\varepsilon_{x\text{'}}+k_{x}^{2}\varepsilon_{y\text{'}}\right)\sin^{2}\alpha+k_{x}k_{y}\left(\varepsilon_{x\text{'}}-\varepsilon_{y\text{'}}\right)\sin2\alpha=\varepsilon_{x\text{'}}\varepsilon_{y\text{'}}k_{0}^{2}$.

According to Eq. (S1), we find that the **condition of the non-reflection or perfect impedance matching (PIM)** is,

$\frac{k_{b,y}}{\varepsilon_{b}}=\frac{\overline{\varepsilon}_{xy}k_{x}+\overline{\varepsilon}_{yy}k_{y}}{\overline{\varepsilon}_{xx}\overline{\varepsilon}_{yy}-\overline{\varepsilon}_{xy}\overline{\varepsilon}_{yx}}$ (S2)

The left term in Eq. (S2) is proportional to the wave impedance $Z_{b}$ of the isotropic dielectric, i.e. $Z_{b}=\frac{E_{x}}{H_{z}}=-\frac{k_{b,y}}{\varepsilon_{b}}\frac{Z_{0}}{k_{0}}$. Here, $E_{x}$ and $H_{z}$ are, respectively, the $x$-component of electric field and $z$-component of magnetic field in the isotropic dielectric. $Z_{0}$ is the vacuum impedance (~377$\Omega$). The right term in Eq. (S2) is proportional to the wave impedance of the TAM:

$Z_{TAM}=-\frac{\overline{\varepsilon}_{xy}k_{x}+\overline{\varepsilon}_{yy}k_{y}}{\overline{\varepsilon}_{xx}\overline{\varepsilon}_{yy}-\overline{\varepsilon}_{xy}\overline{\varepsilon}_{yx}}\frac{Z_{0}}{k_{0}}$ (S3)

According to the dispersion of the TAM, we find out two solutions of $k_{y}$ for a fixed $k_{x}$, that is, $k_{y}^{f}=\frac{-\sqrt{\varepsilon_{x'}}\sqrt{\varepsilon_{y'}}\sqrt{k_{0}^{2}\left(\varepsilon_{x'}\sin^{2}\alpha+\varepsilon_{y'}\cos^{2}\alpha\right)-k_{x}^{2}}+k_{x}\sin2\alpha\left(\varepsilon_{y'}-\varepsilon_{x'}\right)/2}{\varepsilon_{y'}\cos^{2}\alpha+\varepsilon_{x'}\sin^{2}\alpha}$ and $k_{y}^{b}=\frac{\sqrt{\varepsilon_{x'}}\sqrt{\varepsilon_{y'}}\sqrt{k_{0}^{2}\left(\varepsilon_{x'}\sin^{2}\alpha+\varepsilon_{y'}\cos^{2}\alpha\right)-k_{x}^{2}}+k_{x}\sin2\alpha\left(\varepsilon_{y'}-\varepsilon_{x'}\right)/2}{\varepsilon_{y'}\cos^{2}\alpha+\varepsilon_{x'}\sin^{2}\alpha}$, which are related to the forward and backward propagating waves, respectively. By substituting the two solutions into the wave impedance in Eq. (S3), we find that,

$Z_{TAM}\left(k_{x},k_{y}^{f}\right)=-Z_{TAM}\left(k_{x},k_{y}^{b}\right)=\frac{\sqrt{k_{0}^{2}\left(\varepsilon_{x'}\sin^{2}\alpha+\varepsilon_{y'}\cos^{2}\alpha\right)-k_{x}^{2}}}{\sqrt{\varepsilon_{x'}}\sqrt{\varepsilon_{y'}}}\frac{Z_{0}}{k_{0}}$ (S4)

Since the wave impedance in Eq. (S4) is an even function of $k_{x}$, we have $Z_{TAM}\left(k_{x},k_{y}^{f}\right)=Z_{TAM}\left(-k_{x},k_{y}^{f}\right)$ and $Z_{TAM}\left(k_{x},k_{y}^{b}\right)=Z_{TAM}\left(-k_{x},k_{y}^{b}\right)$. Therefore, **for any fixed** $k_{x}$**, the absolute value of the wave impedance is a constant**, that is,

$\left|Z_{TAM}\left(\pm k_{x},k_{y}^{f}\right)\right|=\left|Z_{TAM}\left(\pm k_{x},k_{y}^{b}\right)\right|$ (S5)

Now, by taking dispersions of the isotropic dielectric and the TAM into account, the PIM condition in Eq. (S2) can be rewritten as,

$\varepsilon_{x'}\varepsilon_{y'}k_{0}^{2}\cos^{2}\theta_{i}+\varepsilon_{b}\left[k_{x}^{2}-k_{0}^{2}\left(\varepsilon_{x'}\sin^{2}\alpha+\varepsilon_{y'}\cos^{2}\alpha\right)\right]=0$ (S6)

We assume that the PIM condition is $\varepsilon_{y'}$-independent. The $\varepsilon_{y'}$-independence means that the first derivative of the left term in Eq. (S6) versus $\varepsilon_{y'}$ is zero, thus we have

$\varepsilon_{x'}\cos^{2}\theta_{i}=\varepsilon_{b}\cos^{2}\alpha$ (S7)

Alternatively, we can obtain the condition of $\varepsilon_{y'}$-independence in a physical way. When transmission waves in the TAM propagate along the axis of $\varepsilon_{y'}$, the electric field will polarize along the axis of $\varepsilon_{x'}$. In this situation, the refraction angle is $\alpha$, which is irrelevant to $\varepsilon_{y'}$. Considering the refraction law, we get the relationship between the $\theta_{i}$ and $\alpha$ as,

$\sqrt{\varepsilon_{b}}\sin\theta_{i}=\sqrt{\varepsilon_{x'}}\sin\alpha$ (S8)

By substituting Eq. (S7) or Eq. (S8) into Eq. (S6), we get the same result, that is,

$\left(\varepsilon_{b}-\varepsilon_{x'}\tan^{2}\alpha\right)\left(\varepsilon_{b}-\varepsilon_{x'}\right)=0$ (S9)

**Equation (S9) is the condition of anomalous Brewster effect (ABE), i.e.**$\varepsilon_{y'}$**-independent PIM**. Equation (S9) indicates two classes of solutions to the $\varepsilon_{y'}$-independent PIM:

1. **One is $\varepsilon_{b}-\varepsilon_{x'}\tan^{2}\alpha=0$ (or $\alpha=\arctan\left(\sqrt{\varepsilon_{b}}/\sqrt{\varepsilon_{x'}}\right)$) when $\varepsilon_{b}\neq\varepsilon_{x'}$. Combined with Eq. (S7) or Eq. (S8), we obtain $\theta_{i}=\pm\left(\pi/2-\alpha\right)$.**

When $\theta_{i}=\pi/2-\alpha$, the PIM is similar to the classical Brewster angle effect. In this case, the transmission waves in the TAM propagate along the axis of $\varepsilon_{y'}$, which are also $\varepsilon_{y'}$-independent. However, when $\theta_{i}=-\left(\pi/2-\alpha\right)$, the transmission waves will rely on $\varepsilon_{y'}$ although the PIM is still $\varepsilon_{y'}$-independent. **The same PIM under $\theta_{i}=\pi/2-\alpha$ and $\theta_{i}=-\left(\pi/2-\alpha\right)$ attributes to the reciprocity.**

1. **Another one is $\varepsilon_{b}=\varepsilon_{x'}$, which has no restriction on the value of $\alpha$. Based on Eq. (S7) or Eq. (S8), we obtain $\theta_{i}=\pm\alpha$.**

When $\theta_{i}=\alpha$, we have trivial PIM. In this case, the transmission waves in the TAM propagate along the axis of $\varepsilon_{y'}$, which are also $\varepsilon_{y'}$-independent. However, when $\theta_{i}=-\alpha$, the transmission waves will rely on $\varepsilon_{y'}$ although the PIM is still $\varepsilon_{y'}$-independent. **The same PIM under $\theta_{i}=\alpha$ and $\theta_{i}=-\alpha$ attributes to the reciprocity.**

The above results reveal that for forward propagating waves, we have the same wave impedance when $k_{x}=\pm\sqrt{\varepsilon_{b}}k_{0}\cos\theta_{i}=\pm\sqrt{\varepsilon_{x'}}k_{0}\cos\alpha$, which is $\varepsilon_{y'}$-independent. Combined with Eq. (S5), we further obtain,

$\left|Z_{TAM}\left(\pm\sqrt{\varepsilon_{x'}}k_{0}\sin\alpha,k_{y}^{f}\right)\right|=\left|Z_{TAM}\left(\pm\sqrt{\varepsilon_{x'}}k_{0}\sin\alpha,k_{y}^{b}\right)\right|=\frac{\cos\alpha}{\sqrt{\varepsilon_{x'}}}Z_{0}$ ($\varepsilon_{y'}$-independent) (S10)

Equation (10) indicates **two equal-$\left|Z_{TAM}\right|$ lines of $k_{x}=\pm\sqrt{\varepsilon_{x'}}k_{0}\cos\alpha$ for the TAM with varied** $\varepsilon_{y'}$, as shown in Fig. 1 in the Main Text.

## Complementary discussions of the lossless TAM

First, we present more detailed discussions on the lossless TAM with $\varepsilon_{b}\neq\varepsilon_{x'}$. In order to show the relationship between the incident angle $\theta_{i}$ and the rotation angle $\alpha$ in the realization of $\varepsilon_{y'}$-independent PIM, we take the TAM with $\varepsilon_{x'}=2$ as an example.


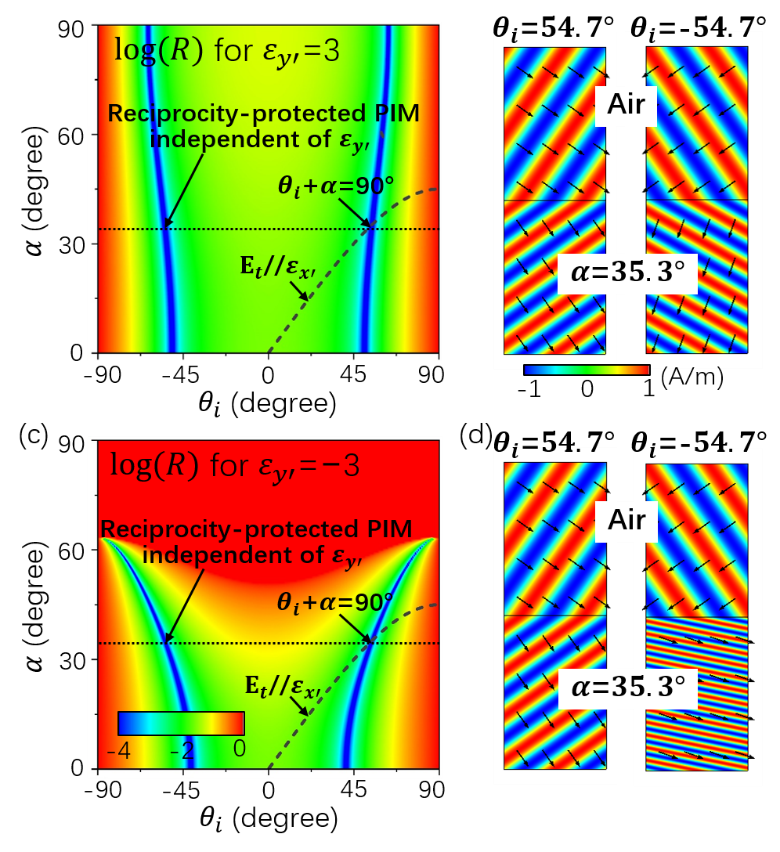


**Figure S1. The relationship between the incident angle and rotation angle in the case of** $\varepsilon_{b}\neq\varepsilon_{x'}$**.** [(a) and (c)] Reflectance at the air-TAM ($\varepsilon_{x'}=2$) interface as functions of the $\theta_{i}$ and $\alpha$. The $\varepsilon_{y'}$ is chosen as (a) 3, (c) -3. The incident waves are of TM polarizations. [(b) and (d)] Distributions of magnetic fields (color) and group velocity (arrows) when a TM polarized wave is incident from air onto the TAM ($\varepsilon_{x'}=2$, $\alpha=35.3^{\circ}$) with (b) $\varepsilon_{y'}=3$, (d) $\varepsilon_{y'}=-3$ under $\theta_{i}=54.7^{\circ}$ (left) and $\theta_{i}=-54.7^{\circ}$ (right).

Figures S1(a) and S1(c) plot the reflectance for TM-polarized waves incident from air onto the TAM with $\varepsilon_{y'}=3$ and $\varepsilon_{y'}=-3$, respectively. The dark blue lines with zero reflectance satisfy Eq. (S6), where the TAM is impedance matched with air. Now, we consider **transmission waves propagating along the axis of $\varepsilon_{y'}$ in the TAM (the electric field $\text{E}_{t}$ is parallel to the axis of $\varepsilon_{x'}$), so that the PIM is $\varepsilon_{y'}$-independent**. In this situation, the relationship between the $\theta_{i}$ and $\alpha$ is determined by Eq. (S8). In Figs. S1(a) and S1(c), we plot the Eq. (S8) as black dashed lines, that intersect the impedance-matched lines (Eq. (S6)) in the $\theta_{i}>0$ space. Although the $\varepsilon_{y'}$ is different, the intersection point in Figs. S1(a) and S1(c) is the same, i.e. $\alpha=\arctan\left(\sqrt{\varepsilon_{b}}/\sqrt{\varepsilon_{x'}}\right)\approx35.3^{\circ}$ and $\theta_{i}=90^{\circ}-\alpha=54.7^{\circ}$, which is related to the $\varepsilon_{y'}$-independent PIM. Interestingly, reciprocity leads to another point of $\varepsilon_{y'}$-independent PIM at $\alpha=\arctan\left(\sqrt{\varepsilon_{b}}/\sqrt{\varepsilon_{x'}}\right)\approx35.3^{\circ}$ and $\theta_{i}=-\left(90^{\circ}-\alpha\right)=-54.7^{\circ}$, as shown in Figs. S1(a) and S1(c). Moreover, we simulate the distributions of magnetic fields (color) and group velocity (arrows) under$\theta_{i}=54.7^{\circ}$ (left) and $\theta_{i}=-54.7^{\circ}$ (right) in Figs. S1(b) and S1(d). When $\theta_{i}=54.7^{\circ}$, we can see the same wave phenomena, i.e. zero reflection and the same refraction angle, despite of the different $\varepsilon_{y'}$. When $\theta_{i}=-54.7^{\circ}$, the reflection is still zero due to the protection of reciprocity. However, it is interesting to see that the varied $\varepsilon_{y'}$ can tune the transmission waves in the TAM, including the refraction angle, group velocity and propagation phase.


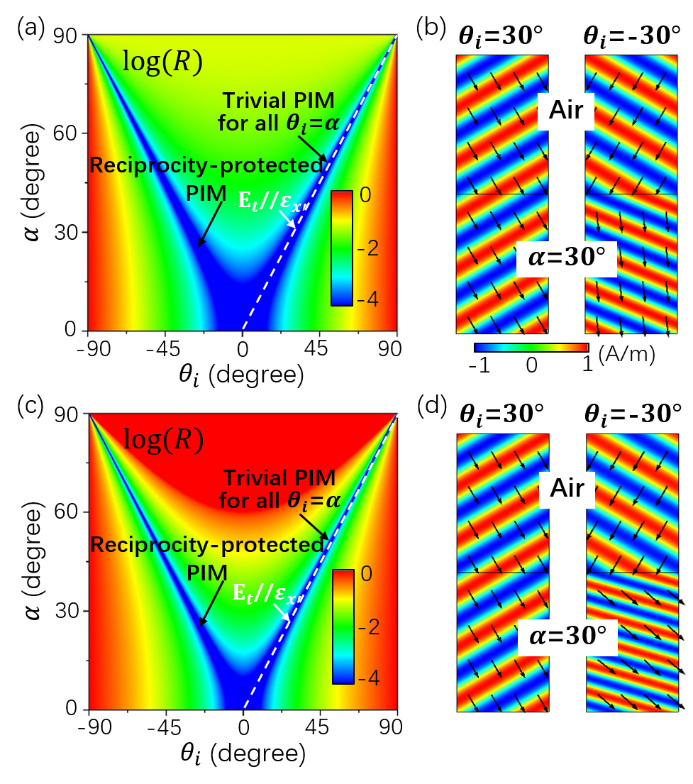


**Figure S2.** **The relationship between the incident angle and rotation angle in the case of** $\varepsilon_{b}=\varepsilon_{x'}$**.** [(a) and (c)] Reflectance at the air-TAM ($\varepsilon_{x'}=1$) interface as functions of the $\theta_{i}$ and $\alpha$ when (a) $\varepsilon_{y'}=3$, (c) $\varepsilon_{y'}=-3$. The incident waves are of TM polarizations. [(b) and (d)] Distributions of magnetic fields (color) and group velocity (arrows) when a TM polarized wave is incident from air onto the TAM ($\varepsilon_{x'}=1$, $\alpha=30^{\circ}$) with (b) $\varepsilon_{y'}=3$, (d) $\varepsilon_{y'}=-3$ under $\theta_{i}=30^{\circ}$ (left) and $\theta_{i}=-30^{\circ}$ (right).

Second, we discuss more about the lossless TAM with $\varepsilon_{b}=\varepsilon_{x'}$. In this case, the condition of ABE in Eq. (S6) is simplified to,

$\left(\varepsilon_{b}-\varepsilon_{y'}\right)\left(\sin^{2}\theta_{i}-\sin^{2}\alpha\right)=0$ (S11)

which indicates the PIM for all $\theta_{i}=\pm\alpha$ when $\varepsilon_{b}\neq\varepsilon_{y'}$.

Without loss of generality, we consider that $\varepsilon_{b}=\varepsilon_{x'}=1$. In Figs. S2(a) and S2(c), the TAM with $\varepsilon_{y'}=3$ and $\varepsilon_{y'}=-3$ are studied as examples. The reflectance at the air-TAM interface is plotted as functions of the $\theta_{i}$ and $\alpha$. The dark blue lines of $\theta_{i}=\pm\alpha$ denote the condition of PIM (i.e. Eq. (S6) or Eq. (S11)). Furthermore, we plot the condition of $\varepsilon_{y'}$-independence (i.e. Eq. (S8)) as white dashed lines, which overlap with the dark blue line in the right side. This indicates that for all $\theta_{i}=\alpha$, we have the $\varepsilon_{y'}$-dependent PIM, and there is no restriction on the value of the $\alpha$. Considering the reciprocity, we have the same $\varepsilon_{y'}$-dependent PIM for all $\theta_{i}=-\alpha$. For verification, we take the TAM with $\alpha=30^{\circ}$ as an example. Figures S2(b) and S2(d) show the distributions magnetic fields (color) and group velocity (arrows) under $\theta_{i}=30^{\circ}$ (left) and $\theta_{i}=-30^{\circ}$ (right), showing the $\varepsilon_{y'}$-independent zero reflection under $\theta_{i}=\pm30^{\circ}$, and the $\varepsilon_{y'}$-controlled transmission waves under $\theta_{i}=-30^{\circ}$.

## Ultra-broadband reflection-less positive and negative refraction, and experimental verification

In this section, we show that the $\varepsilon_{y'}$-independent PIM and $\varepsilon_{y'}$-controlled flexible refraction can be simultaneously obtained. In this way, we can realize both reflection-less positive and negative refraction. Figure S3(a) presents the EFCs of air (black dashed lines) and the TAM ($\varepsilon_{x'}=1$) with varied $\varepsilon_{y'}$ (blue solid lines). We know that when TM-polarized waves are incident from air onto the TAM under $\theta_{i}=\pm\alpha$, we have the $\varepsilon_{y'}$-independent PIM. The green arrows in Fig. S3(a) denote wave vectors under $\theta_{i}=\pm\alpha$. Considering the conservation of wave-vector component parallel to the air-TAM surface, we can find out the direction of group velocity $\text{v}_{g}$ of transmission waves in the TAM, which is normal to the EFCs (red arrows). It is seen from Fig. S3(a) that the direction of $\text{v}_{g}$ is $\varepsilon_{y'}$-independent under $\theta_{i}=\alpha$ (see the point I). However, interestingly, under $\theta_{i}=-\alpha$, the direction of $\text{v}_{g}$ changes as the variation of $\varepsilon_{y'}$, and both reflection-less positive and negative refraction can be obtained (see the point II).

**
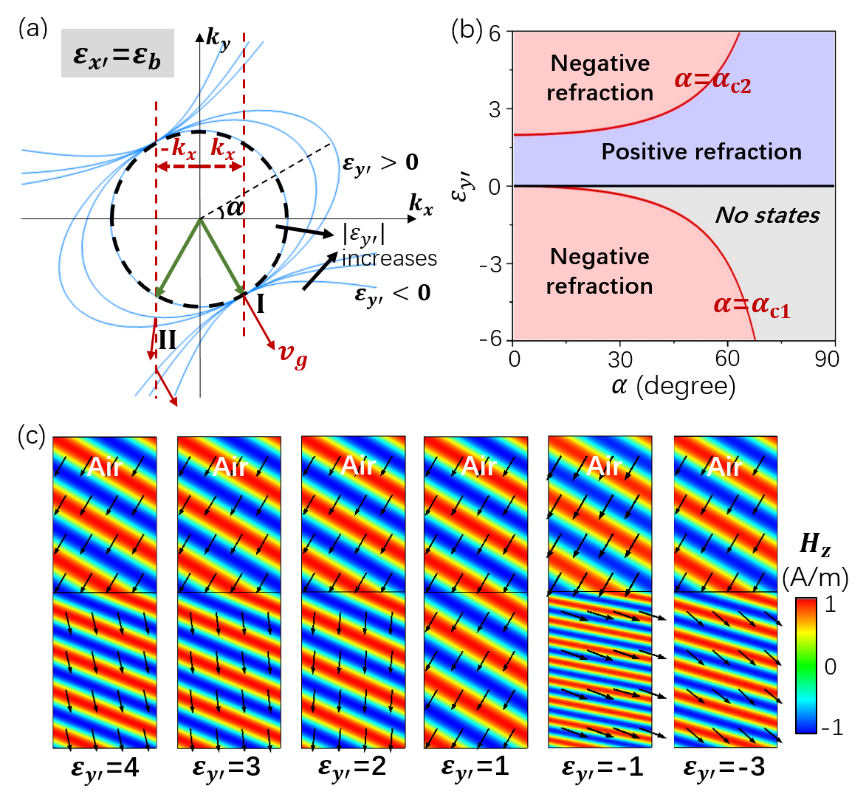
**

**Figure S3. Simultaneous realization of $\varepsilon_{y'}$-independent PIM and $\varepsilon_{y'}$-controlled flexible refraction.** (a) EFCs of air (black dashed lines) and the TAM ($\varepsilon_{x'}=1$) with varied $\varepsilon_{y'}$ (blue solid lines). The red arrows denote directions of group velocity of transmission waves in the TAM. (b) Phase diagram of reflection-less refraction in the $\alpha\text{-}\varepsilon_{y'}$ space under $\theta_{i}=-\alpha$. (c) Simulated distributions of magnetic fields (color) and group velocity (arrows) for TM-polarized waves incident from air onto the TAM ($\varepsilon_{x'}=1$, $\alpha=30^{\circ}$) with different $\varepsilon_{y'}$ under $\theta_{i}=-30^{\circ}$.

Figure S3(b) shows the phase diagram of reflection-less positive and negative refraction in the $\alpha\text{-}\varepsilon_{y'}$ space under $\theta_{i}=-\alpha$. It is seen that when $\varepsilon_{y'}<0$, we generally have reflection-less negative refraction unless $\alpha>\alpha_{c1}$. Here $\alpha_{c1}$ is a critical angle determined by

$\varepsilon_{y'}=-\varepsilon_{x'}\tan^{2}\alpha_{c1}$ (S12)

When $\alpha=\alpha_{c1}$, one of the asymptotes of hyperbolic EFC of the TAM will overlap with the $k_{y}$ axis. Once $\alpha>\alpha_{c1}$, there will be no states supporting the forward propagating waves. Physically, it is not strictly correct, because wave vectors cannot be infinitely large and material losses are inevitable in practical hyperbolic media. As long as a small amount of material loss is considered, there will exist states supporting the forward propagating waves when $\alpha>\alpha_{c1}$.

On the other hand, when $\varepsilon_{y'}>0$, phase transition from reflection-less positive refraction to reflection-less negative refraction, or vice versa, will occur at the critical rotation angle $\alpha_{c2}$:

$\varepsilon_{y'}=\frac{1}{2}\varepsilon_{x'}\left(3+\cos2\alpha_{c2}\right)\sec^{2}\alpha_{c2}$ (S13)

which is related to zero slope of the TAM EFC at the point II.

For demonstrations, we simulate the wave propagation from air onto the TAM with $\varepsilon_{x'}=1$ and $\alpha=30^{\circ}$. Figure S3(c) displays the distributions of magnetic fields (color) and group velocity (arrows) under $\theta_{i}=-30^{\circ}$ when $\varepsilon_{y'}$ is chosen as 4, 3, 2, 1, -1 and -3 for examples. Apparently, there are no reflections in all examples. Moreover, we see that the direction of group velocity of transmission waves in the TAM can be efficiently manipulated by $\varepsilon_{y'}$, thus demonstrating **the simultaneous realization of the $\varepsilon_{y'}$-independent PIM and $\varepsilon_{y'}$-controlled reflection-less positive and negative refraction**.


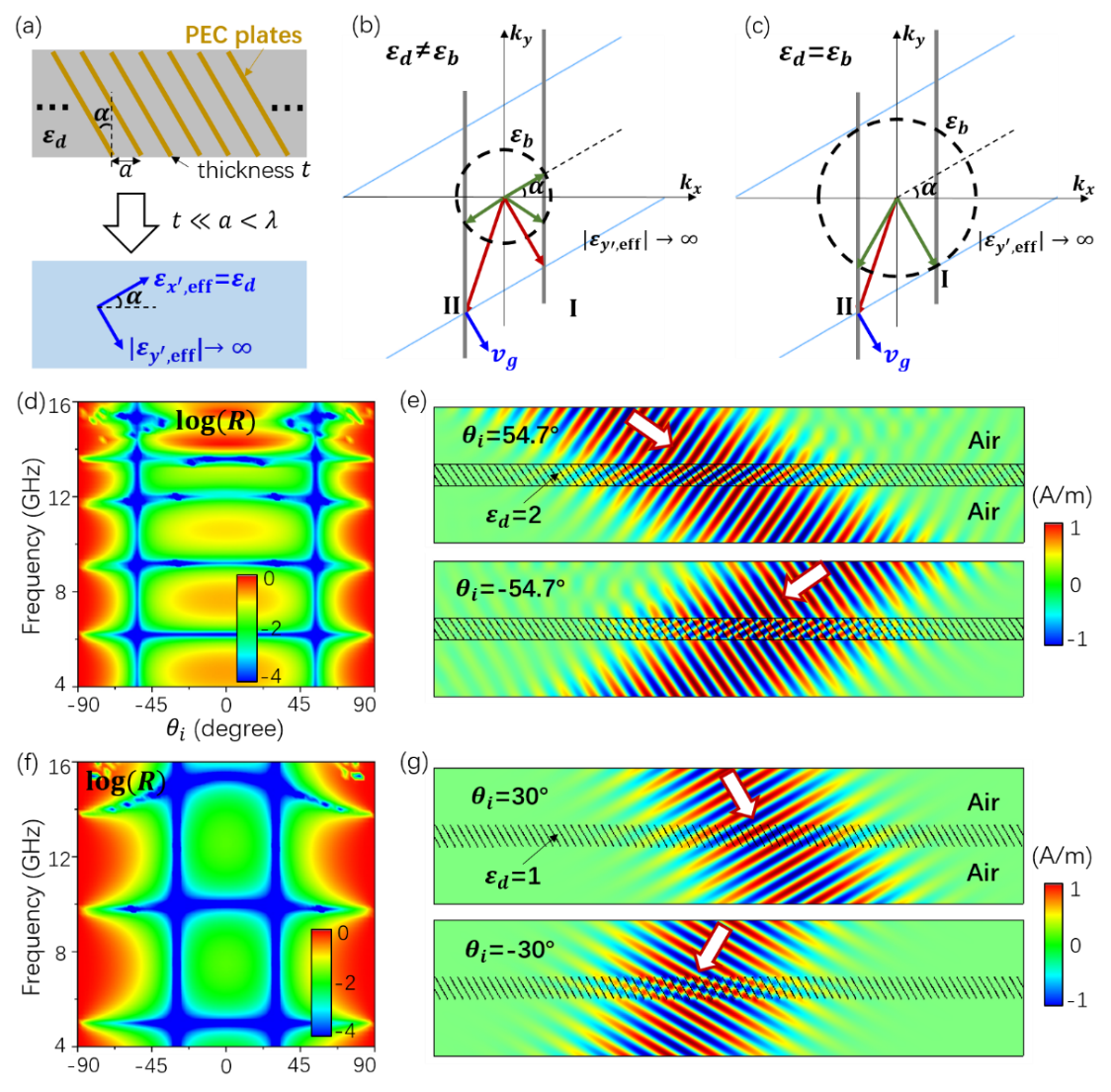


**Figure S4. Theoretical investigation of ultra-broadband reflection-less negative refraction by using tilted PEC plate arrays.** (a) Illustrations of a tilted PEC plate array in an isotropic dielectric of $\varepsilon_{d}$ (upper) and the corresponding effective TAM (lower). [(b) and (c)] The EFCs of a dielectric of $\varepsilon_{b}$ (dashed lines) and the effective TAM (solid lines) for the cases of (b) $\varepsilon_{d}\neq\varepsilon_{b}$ and (c) $\varepsilon_{d}=\varepsilon_{b}$. The green and red arrows denote the incident/reflection and transmission beams, respectively. The blue arrows show the direction of group velocity of the transmission waves in the TAM. [(d) and (f)] Reflectance as functions of the $\theta_{i}$ and working frequency when a TM-polarized wave is incident from air onto the tilted PEC plate array with (d) $\varepsilon_{d}=2$ and $\alpha$=35.3°, (f) $\varepsilon_{d}=1$ and $\alpha$=30°. [(e) and (g)] Simulated magnetic field-distributions at 10 GHz for the cases of (e) $\varepsilon_{d}=2$ and $\alpha=35.3^{\circ}$ under $\theta_{i}=\pm54.7^{\circ}$, (g) $\varepsilon_{d}=1$ and $\alpha=30^{\circ}$ under $\theta_{i}=\pm30^{\circ}$.

Next, we demonstrate the ultra-broadband reflection-less negative refraction by using tilted perfect electric conductor (PEC) plates in both simulations and microwave experiments. The model we studied is illustrated by the upper inset in Fig. S4(a). Ultrathin PEC plates with a rotation angle of $\alpha$ are periodically aligned in an isotropic dielectric (relative permittivity $\varepsilon_{d}$). The separation distance between two adjacent PEC plates is $a$, which is much larger than the thickness of the PEC plates $t$ (i.e. $a\gg t$), but smaller than the wavelength $\lambda$ in the dielectric (i.e. $a<\lambda$). Under this circumstance, we can approximately homogenize the tilted PEC plate array as an effective TAM with

$\varepsilon_{x'\text{,eff}}\approx\varepsilon_{d}$ and $\left|\varepsilon_{y'\text{,eff}}\right|\to\infty$ (S14)

In this situation, the wave impedance of the effective TAM (i.e. Eq. (S4)) turns to be,

$Z_{TAM}\approx\frac{\cos\alpha}{\sqrt{\varepsilon_{d}}}Z_{0}$ (S15)

which indicates a **frequency-independent wave impedance**. Based on this unique feature, ultra-broadband reflection-less negative reflection can be realized, as we shall demonstrate as follows.

Figure S4(b) shows the EFCs of the isotropic dielectric (dashed lines) and the effective TAM with $\left|\varepsilon_{y'\text{,eff}}\right|\to\infty$ (solid lines) for the case of $\varepsilon_{d}\neq\varepsilon_{b}$. It is seen that when $\theta_{i}=\pi/2-\alpha$ and $\alpha=\arctan\sqrt{\varepsilon_{b}/\varepsilon_{d}}$, the TAM operates at the point I, and the reflection beam (green arrows) is perpendicular to the refraction beam (red arrows). This would lead to the PIM similar to Brewster effect. We note that **such PIM is frequency-independent, because both wave impedances of the dielectric and the effective TAM are frequency-independent**. Considering the reciprocity principle, the frequency-independent PIM reserves under $\theta_{i}=-\left(\pi/2-\alpha\right)$ (i.e. the TAM operates at the point II). Interestingly, in this case, we have negative refraction, as illustrated by the blue arrows in Fig. S4(b). Thus, ultra-broadband reflection-less negative refraction can be attained. Similarly, in the case of $\varepsilon_{d}=\varepsilon_{b}$, due to the frequency-independent PIM under $\theta_{i}=\alpha$ and the reciprocity, we can also get the frequency-independent PIM and negative refraction under $\theta_{i}=-\alpha$, as illustrated in Fig. S4(c).

For verification, we first study the case of $\varepsilon_{d}\neq\varepsilon_{b}$ by assuming a tilted PEC plate array ($a$=10 mm, $\alpha$=35.3°) embedded in an isotropic dielectric of $\varepsilon_{d}=2$. The array has a thickness of $d$=30 mm, and is placed in the background of air (i.e. $\varepsilon_{b}=1$). Figure S4(d) presents the reflectance as functions of the $\theta_{i}$ and working frequency under the illumination of a TM-polarized wave, showing frequency-independent zero reflection under $\theta_{i}=\pm54.7^{\circ}$. In Fig. S4(e), the simulated magnetic field-distributions under $\theta_{i}=54.7^{\circ}$ (upper) and $\theta_{i}=-54.7^{\circ}$ (lower) at 10 GHz clearly demonstrate the zero reflection and negative refraction at $\theta_{i}=-54.7^{\circ}$. Second, we study the case of $\varepsilon_{d}=\varepsilon_{b}=1$. The tilted PEC plate array is characterized by $a$=10 mm, $d$=30 mm and $\alpha$=30°. The calculated reflectance in Fig. S4(f) shows the frequency-independent zero reflection at $\theta_{i}=\pm30^{\circ}$. Moreover, the simulated magnetic field-distributions at 10 GHz in Fig. S4(g) show obvious negative refraction under $\theta_{i}=-30^{\circ}$. **Apparently, these results demonstrate the ultra-broadband reflection-less negative refraction by using a tilted PEC plate array.**


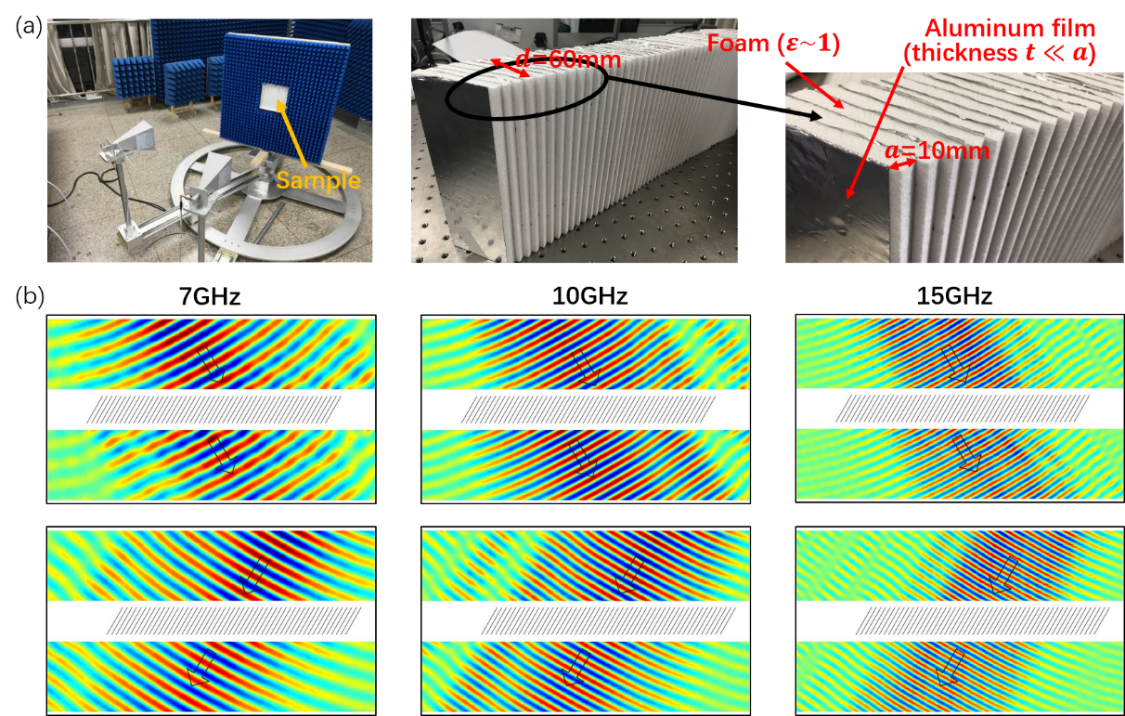


**Figure S5. Experimental observation of ultra-broadband reflection-less negative refraction.** (a) Photographs of the experimental setup and fabricated sample composed of alternative foam and aluminum films with $\alpha$=30°, $a$=10 mm and $d$=60 mm. (b) Measured electric fields under $\theta_{i}=30^{\circ}$ (upper) and $\theta_{i}=-30^{\circ}$ (lower) at 7 GHz (left), 10 GHz (middle) and 15 GHz (right).

We have also performed microwave experiments to demonstrate such ultra-broadband reflection-less negative refraction. Figure S5(a) presents the photographs of the experimental setup and fabricated sample. The sample is composed of alternative foam (relative permittivity ~1) and aluminum films (near PEC films) with $\alpha$=30°, $a$=10 mm and $d$=60 mm. The thickness of the aluminum films is ultrathin, much smaller than $a$. Figure S5(b) displays the measured near-field electric fields under $\theta_{i}=30^{\circ}$ (upper) and $\theta_{i}=-30^{\circ}$ (lower) at 7 GHz (left), 10 GHz (middle) and 15 GHz (right). The details of the experimental setup and measurement methods are discussed in the following Section 8. **The experimental results clearly confirm the ultra-broadband PIM under $\theta_{i}=\pm\alpha$, as well as ultra-broadband reflection-less negative refraction under $\theta_{i}=-\alpha$.**

## Loss-induced breakdown of the Brewster effect in isotropic dielectrics

As a comparison of loss-independent Brewster effect in TAM, here we show that the Brewster effect breaks down immediately when loss is introduced to isotropic dielectrics. Figures S6(a) and 6(b) plot reflectance $\log\left(R\right)$ as functions of incident angle $\theta_{i}$ and $\gamma\left(\omega\right)$ for TM-polarized waves incident onto isotropic dielectric with $\varepsilon=2+\gamma\left(\omega\right)$ and $\varepsilon=1+\gamma\left(\omega\right)$, respectively. **Clearly, the Brewster effect can no longer be maintained and dramatic reflection occurs as long as the loss is introduced.**


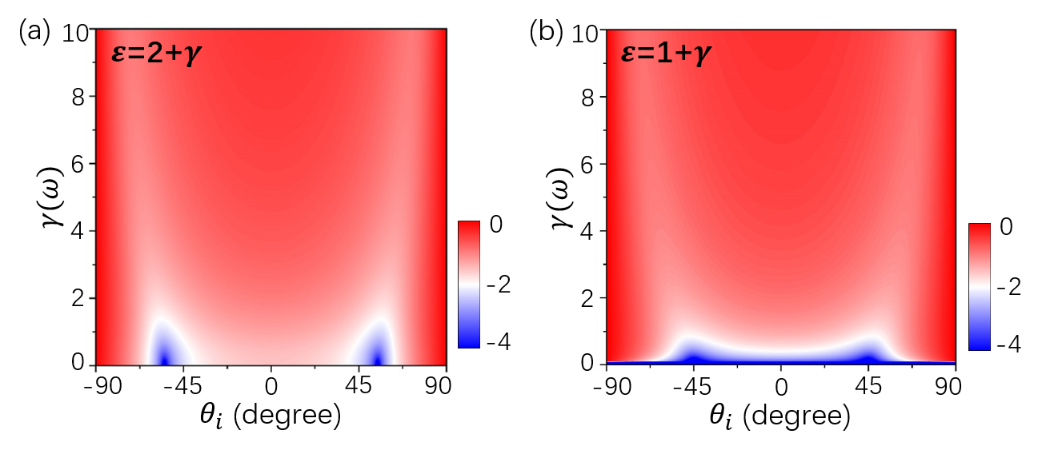


**Figure S6. Loss-induced breakdown of the Brewster effect in isotropic dielectrics.** Calculated reflectance $\log\left(R\right)$ as functions of incident angle $\theta_{i}$ and $\gamma\left(\omega\right)$ for TM-polarized waves incident onto isotropic dielectric with (a) $\varepsilon=2+\gamma\left(\omega\right)$, (b) $\varepsilon=1+\gamma\left(\omega\right)$.

## ABE and perfect absorption in general TAM with material loss

In the Main Text, we have demonstrated the ABE and perfect absorption with tilted CF arrays, whose effective parameters satisfy $\varepsilon_{x'}=\mathrm{Re}\left(\varepsilon_{y'}\right)$. In this section, we discuss the ABE and perfect absorption in general TAM with $\varepsilon_{x'}\neq\mathrm{Re}\left(\varepsilon_{y'}\right)$.

**
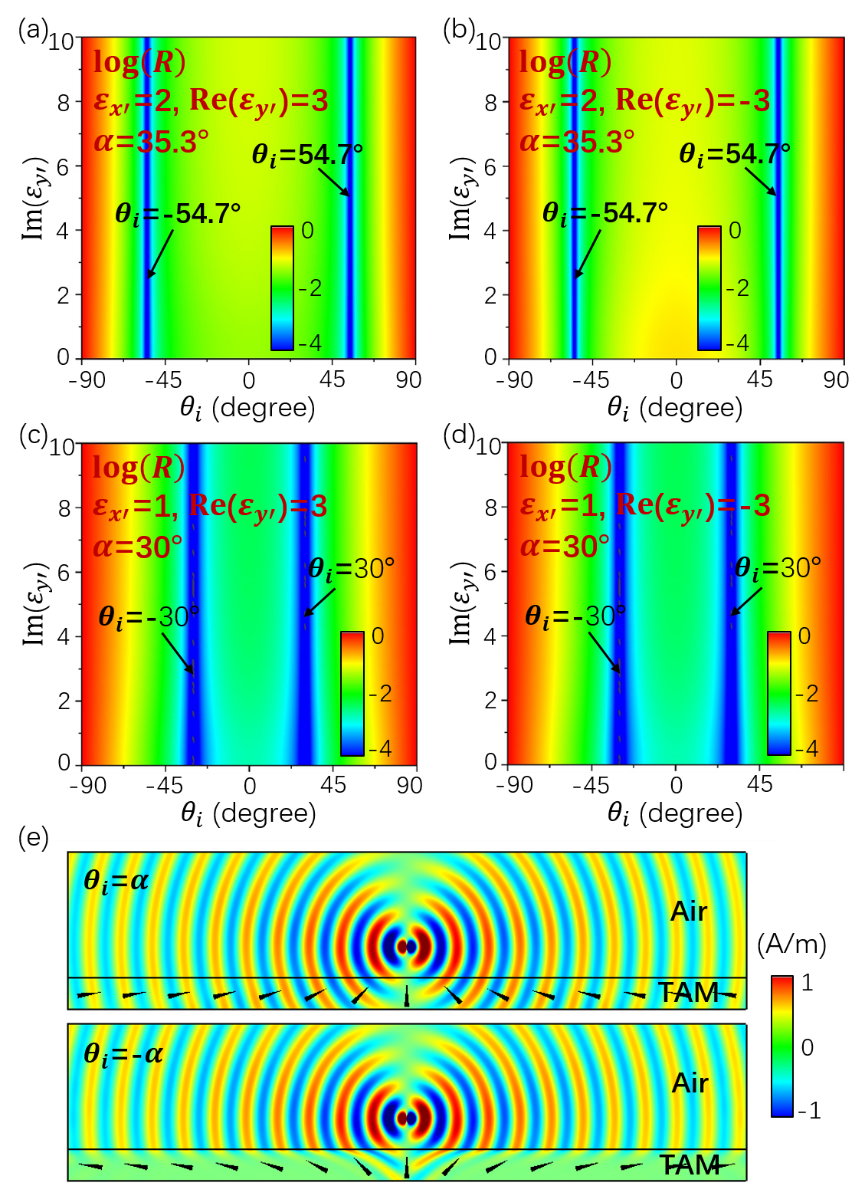
**

**Figure S7. ABE and perfect absorption in general TAM with $\varepsilon_{x'}\neq\mathrm{Re}\left(\varepsilon_{y'}\right)$.** [(a)-(d)] Reflectance of TM-polarized waves incident from air onto the TAM with (a) $\varepsilon_{x'}=2$, $\mathrm{Re}\left(\varepsilon_{y'}\right)=3$, $\alpha=35.3^{\circ}$, (b) $\varepsilon_{x'}=2$, $\mathrm{Re}\left(\varepsilon_{y'}\right)=-3$, $\alpha=35.3^{\circ}$, (c) $\varepsilon_{x'}=1$, $\mathrm{Re}\left(\varepsilon_{y'}\right)=3$, $\alpha=30^{\circ}$, (d) $\varepsilon_{x'}=1$, $\mathrm{Re}\left(\varepsilon_{y'}\right)=-3$, $\alpha=30^{\circ}$. (e) Simulated magnetic field-distributions when a TM-polarized dipole source is placed above an inhomogeneous TAM slab with fixed $\varepsilon_{x'}=1$, $\varepsilon_{y'}=3+3.33i$ and thickness $d$=30 mm at 10 GHz. The orientation of $\varepsilon_{y'}$ of the TAM (black arrows) is engineered, so that the condition of $\theta_{i}=\alpha$ (upper) or $\theta_{i}=-\alpha$ (lower) is satisfied everywhere.

Here, we take the TAM with $\varepsilon_{x'}=2$, $\mathrm{Re}\left(\varepsilon_{y'}\right)=\pm3$, $\alpha=35.3^{\circ}$ in Figs. S7(a) and S7(b), and the TAM with $\varepsilon_{x'}=1$, $\mathrm{Re}\left(\varepsilon_{y'}\right)=\pm3$, $\alpha=30^{\circ}$ in Figs. S7(c) and S7(d) as examples. Figures S7(a)-S7(d) show the reflectance as functions of the $\theta_{i}$ and $\mathrm{Im}\left(\varepsilon_{y'}\right)$ when TM-polarized waves incident from air onto the TAM. Apparently, $\mathrm{Im}\left(\varepsilon_{y'}\right)$-independent zero reflection is seen at $\theta_{i}=\pm54.7^{\circ}$ (or $\theta_{i}=\pm30^{\circ}$) for the TAM with $\varepsilon_{x'}=2$ (or $\varepsilon_{x'}=1$), demonstrating the ABE, i.e. $\varepsilon_{y'}$-independent PIM, in the existence of material loss.

Furthermore, in Fig. S7(e), we perform simulations by illuminating an inhomogeneous TAM slab ($\varepsilon_{x'}=1$, $\varepsilon_{y'}=3+3.33i$, thickness $d$=30 mm) with a TM-polarized dipole source at 10 GHz. The orientation of $\varepsilon_{y'}$ of the TAM (black arrows) is engineered so that the condition of $\theta_{i}=\alpha$ (upper) or $\theta_{i}=-\alpha$ (lower) is satisfied everywhere. It is seen that in both cases of $\theta_{i}=\pm\alpha$, there are no reflection waves, demonstrating the omnidirectional PIM. Interestingly, when $\theta_{i}=-\alpha$, rapid attenuation of transmission waves in the TAM is observed, demonstrating the omnidirectional perfect absorption.

**The above results clearly demonstrate that PIM is completely independent of both the real and imaginary parts of $\varepsilon_{y'}$, and the perfect absorption can be realized in general TAM with $\varepsilon_{x'}\neq\mathrm{Re}\left(\varepsilon_{y'}\right)$.**

## Extraordinary ultrathin perfect absorbers by TAM with hyperbolic dispersions

In this section, we first study the attenuation rate of transmission waves in TAM with material losses, and then demonstrate an extraordinary kind of ultrathin perfect absorbers by TAM with hyperbolic dispersions.


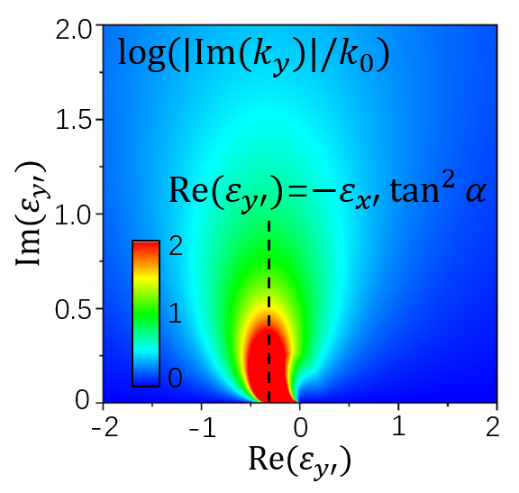


**Figure S8. Attenuation rate of transmission waves in TAM with material losses.** $\log\left(\left|\mathrm{Im}\left(k_{y}\right)\right|/k_{0}\right)$ as functions of the $\mathrm{Re}\left(\varepsilon_{y'}\right)$ and $\mathrm{Im}\left(\varepsilon_{y'}\right)$. $k_{y}$ is the $y$-component of wave vector of transmission waves in the TAM ($\varepsilon_{x'}=1$, $\alpha=30^{\circ}$).

In Fig. S8, we take the TAM with $\varepsilon_{x'}=1$ and $\alpha=30^{\circ}$ as an example. $\log\left(\left|\mathrm{Im}\left(k_{y}\right)\right|/k_{0}\right)$ of transmission waves in the TAM is calculated as functions of the $\mathrm{Re}\left(\varepsilon_{y'}\right)$ and $\mathrm{Im}\left(\varepsilon_{y'}\right)$, showing extraordinary large $\mathrm{Im}\left(k_{y}\right)$ at

$\mathrm{Re}\left(\varepsilon_{y'}\right)=-\varepsilon_{x'}\tan^{2}\alpha$ (S16)

Equation (S16) indicates that $\varepsilon_{x'}$ and $\mathrm{Re}\left(\varepsilon_{y'}\right)$ have opposite signs, that is, the TAM possesses a hyperbolic dispersion in the absence of material losses. Compared with Eq. (S12), we find that **the extraordinary large $\mathrm{Im}\left(k_{y}\right)$ occurs when one of the asymptotes of hyperbolic EFC overlaps with the $k_{y}$ axis. Intriguingly, when $\mathrm{Im}\left(\varepsilon_{y'}\right)\approx0$, infinitely large $\mathrm{Im}\left(k_{y}\right)$ can be obtained, revealing a unique kind of ultrathin perfect absorbers**. Moreover, it is seen that **the increase of $\mathrm{Im}\left(\varepsilon_{y'}\right)$ will lead to the decrease of $\mathrm{Im}\left(k_{y}\right)$, showing an extraordinary absorption behavior**.

For verification, Fig. S9(a) displays magnetic field-distributions when a TM-polarized wave is incident from air onto the TAM with $\varepsilon_{x'}=1$, $\varepsilon_{y'}=-3+0.1i$ and $\alpha=60^{\circ}$, showing non-absorption under $\theta_{i}=60^{\circ}$ (left) and perfect absorption within an ultrathin thickness under $\theta_{i}=-60^{\circ}$ (right). For comparison, we increase the $\mathrm{Im}\left(\varepsilon_{y'}\right)$, so that $\varepsilon_{y'}=-3+10i$ in Fig. S9(b). The zero absorption under $\theta_{i}=60^{\circ}$ (left) and perfect absorption under $\theta_{i}=-60^{\circ}$ (right) are not changed. But the transmission waves in the TAM decay much more slowly than those in the TAM with $\varepsilon_{y'}=-3+0.1i$.


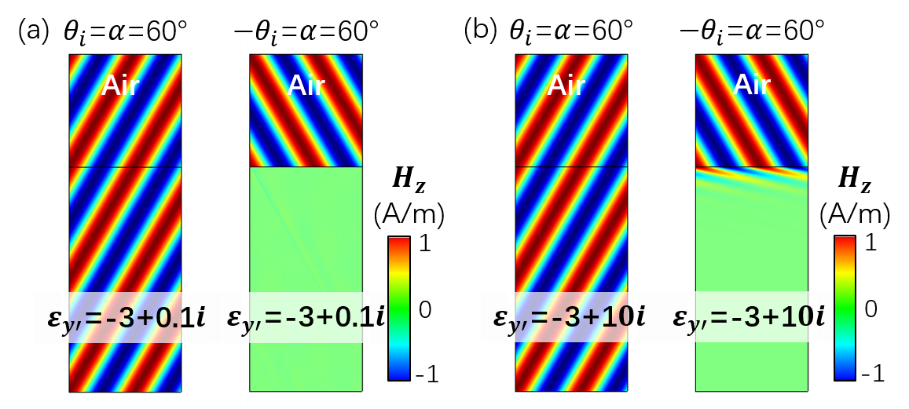


**Figure S9. Extraordinary perfect absorption by TAM with hyperbolic dispersions.** [(a) and (b)] Magnetic field-distributions when a TM-polarized wave is incident from air onto the TAM ($\varepsilon_{x'}=1$, $\alpha=60^{\circ}$) with (a) $\varepsilon_{y'}=-3+0.1i$, (b) $\varepsilon_{y'}=-3+10i$ under $\theta_{i}=60^{\circ}$ (left) and $\theta_{i}=-60^{\circ}$ (right).

**These results reveal that the thickness of the TAM absorber can be pushed to zero as the material loss (i.e. $\mathrm{Im}\left(\varepsilon_{y'}\right)$) tends to be zero, which is totally different from the common understanding that thinner absorbers need larger material losses to maintain the same absorption. Actually,** **such extraordinary absorption originates from the enhanced electric fields normal to the air-TAM interface, which increase rapidly as $\mathrm{Im}\left(\varepsilon_{y'}\right)$ decreases**.

It is worth noting that the physical origin of the extraordinary absorption by the ultrathin TAM is very similar to the extraordinary absorption by zero-index media^1,2^. In addition, we notice that the absorption by the tilted (or asymmetric) hyperbolic media has been discussed in^3^. In these works, material loss is introduced to both $\varepsilon_{x'}$ and $\varepsilon_{y'}$, therefore, *the PIM is destroyed*. As a consequence, only when the material loss is small, can the near-perfect absorption be obtained. As long as the material loss is increased, the absorption will decrease and dramatic reflections will occur due to impedance mismatch.

## Effective medium model of tilted CF array and ABE from dc to the GHz regime

In this section, we discuss the effective medium model of tilted conductive film (CF) array from dc to the GHz regime. The model we studied is illustrated by the left inset in Fig. S10. The CFs with a rotation angle of $\alpha$ are periodically aligned in an isotropic dielectric background (relative permittivity $\varepsilon_{d}$). The separation distance between two adjacent two CFs is $a$, which is much larger than the thickness $t$ of the CFs, i.e. $t\ll a$. In low frequency regime, the relative permittivity of the CFs can be expressed as $\varepsilon_{CF}=1+i\frac{Z_{0}/R_{s}}{k_{0}t}$, where $R_{s}$ is sheet resistance of the CFs.

We assume that the separation distance $a$ is smaller than the wavelength $\lambda$ in the background dielectric, i.e. $a<\lambda$. Under this circumstance, we can approximately homogenize the tilted CF array based on classical Maxwell-Garnett theory^4^ as an effective TAM (see the right inset in Fig. S10) with

$\varepsilon_{x'\text{,eff}}=\frac{\varepsilon_{CF}\varepsilon_{d}a\cos\alpha}{\varepsilon_{CF}\left(a\cos\alpha-t\right)+\varepsilon_{d}t}\approx\varepsilon_{d}$ and $\varepsilon_{y'\text{,eff}}=\frac{\varepsilon_{d}\left(a\cos\alpha-t\right)+\varepsilon_{CF}t}{a\cos\alpha}\approx\varepsilon_{d}+i\gamma\left(\omega\right)$ (S17)

where $\gamma\left(\omega\right)=\frac{Z_{0}/R_{s}}{k_{0}a\cos\alpha}$. Equation (S17) shows that $\varepsilon_{x'\text{,eff}}=\mathrm{Re}\left(\varepsilon_{y'\text{,eff}}\right)$ is frequency-independent, while $\mathrm{Im}\left(\varepsilon_{y'\text{,eff}}\right)$ relies on the working frequency.


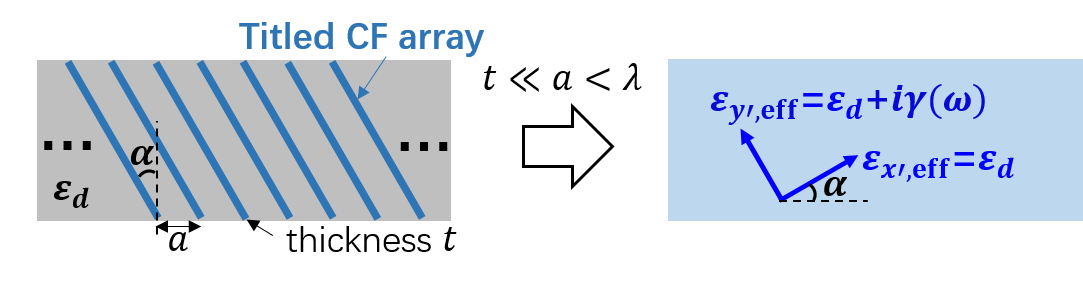


**Figure S10. Effective medium model of tilted CF array.** Illustrations of a tilted CF array in an isotropic dielectric background (left) and the corresponding effective medium model (right).

It is noteworthy that the Eq. (S17) is derived based on quasi-static limit, therefore, **there is no lower frequency limit**. Actually, with the titled CF array, we can realize PIM from the quasi-static limit to the GHz regime. As an example, we consider a tilted CF array with $a$=5 mm, $\varepsilon_{d}=1$ and $\alpha=30^{\circ}$. Figure S11 presents the reflection coefficient at the interface of free space and the effective medium of the tilted CF array as functions of the incident angle and working frequency. The working frequency varies from dc to the KHz, MHz and GHz regimes. **The results clearly show that the PIM preserves from dc to the GHz regime, indicating the ultra-broadband ABE.**


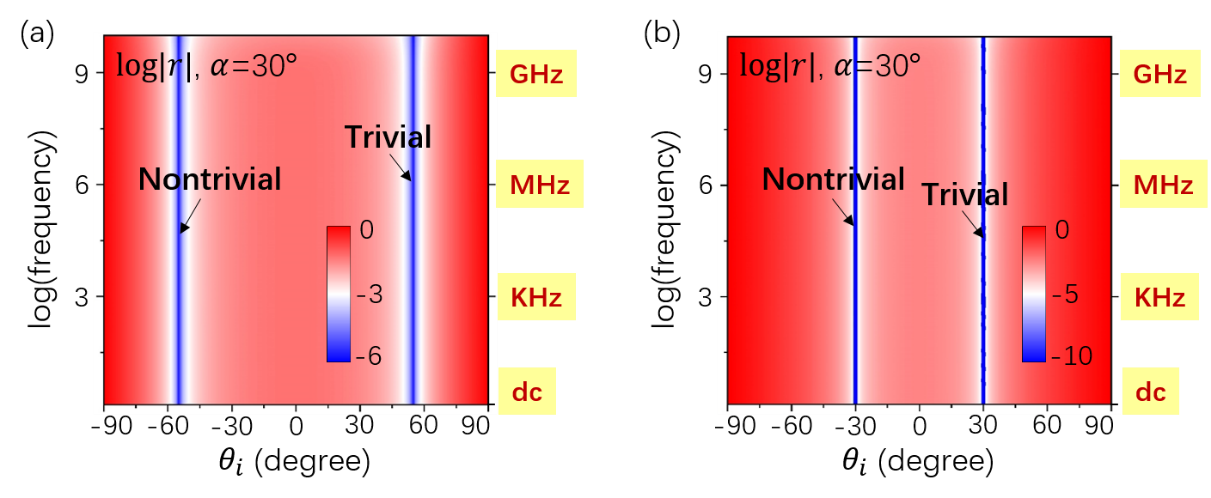


**Figure S11. ABE from dc to the GHz regime.** [(a) and (b)] Reflection coefficient at the interface of free space and the effective medium of the tilted CF array as functions of the incident angle and working frequency. The relevant parameters of the tilted CF array are (a) $a$=5 mm, $\varepsilon_{d}=2$ and $\alpha=35.3^{\circ}$, (b) $a$=5 mm, $\varepsilon_{d}=1$ and $\alpha=30^{\circ}$. The working frequency varies from dc to the GHz regime.

On the other hand, the upper frequency limit is determined by the separation distance $a$. The operating wavelength should satisfy $\lambda>a$ to guarantee the validity of the effective medium model. More specifically, we need to make sure that there are no diffraction waves, which requires $\frac{2\pi}{a}>\sqrt{\varepsilon_{b}}\frac{2\pi}{\lambda_{0}}+\sqrt{\varepsilon_{b}}\frac{2\pi}{\lambda_{0}}\sin\theta_{i}$ and $\frac{2\pi}{a}>\sqrt{\varepsilon_{d}}\frac{2\pi}{\lambda_{0}}+\sqrt{\varepsilon_{b}}\frac{2\pi}{\lambda_{0}}\sin\theta_{i}$, leading to

$a<\frac{\lambda_{0}}{\sqrt{\varepsilon_{b}}\left(1+\sin\theta_{i}\right)}$ and $a<\frac{\lambda_{0}}{\sqrt{\varepsilon_{d}}+\sqrt{\varepsilon_{b}}\sin\theta_{i}}$ (S18)

where $\lambda_{0}$ is the wavelength in free space.

**Since the separation distance of fabricated experimental samples is 5~10 mm, the upper working frequency limit is around 20 GHz, as implied by Eq. (S18). It is noteworthy that such upper frequency limit can be raised to higher frequency regimes (e.g. THz, infrared and optical regimes) by reducing the separation distance.**


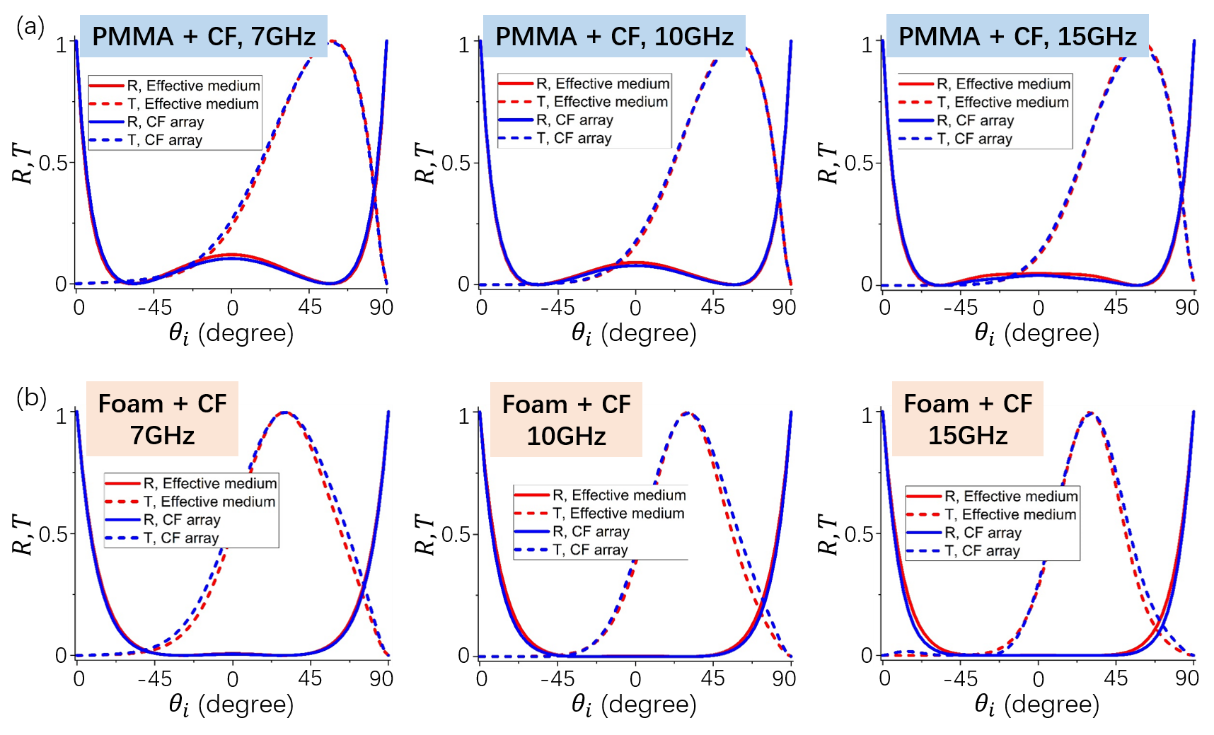


**Figure S12. Verification of the validity of effective medium models of experimental tilted CF array samples in the GHz regime.** [(a) and (b)] Reflectance and transmittance of the experimental sample (blue lines) and the corresponding effective medium (red lines) at 7 GHz (left), 10 GHz (middle) and 15 GHz (right). In (a), the sample consisting of PMMA and ITO films is the same as that in Fig. 3. In (b), the sample consisting of foam and ITO films is same as that in Fig. 4.

Next, we check the validity of effective medium models of the experimental tilted CF array samples at studied frequencies 7 GHz, 10 GHz and 15 GHz (i.e. the samples in Figs. 3 and 4 in the Main Text). First, we consider the sample consisting of PMMA and ITO films (the same sample in Fig. 3). The relevant parameters are $a$=5 mm, $\varepsilon_{b}=1$, $\varepsilon_{d}=2.6$ and $\theta_{i}=58.2^{\circ}$, indicating an upper frequency limit of 24.4 GHz. Therefore, in our experiments, frequencies below 16 GHz are studied. In Fig. S12(a), we compare the reflectance and transmittance for TM-polarized waves incident from air onto the experimental sample and the corresponding effective medium, showing quite good coincidence at 7 GHz (left), 10 GHz (middle) and 15 GHz (right). Second, we consider the sample consisting of foam and ITO films (the same sample in Fig. 4), whose relevant parameters are $a$=10 mm, $\varepsilon_{b}=\varepsilon_{d}=1$ and $\theta_{i}=30^{\circ}$. This indicates an upper frequency limit of 20 GHz. Figure S12(b) shows the reflectance and transmittance at 7 GHz (left), 10 GHz (middle) and 15 GHz (right), also showing quite good coincidence between the experimental sample and the effective medium. **These results clearly demonstrate the validity of effective medium models of experimental samples at working frequencies (<16 GHz).**

## Optimal sheet resistance analysis, details of experimental samples and further experimental results

In this section, we first investigate the influences of sheet resistance $R_{s}$ in the attenuation rate of transmission waves in the TAM, and try to find out the optimal $R_{s}$ to obtain the largest attenuation rate, so that the absorbers can be as thin as possible. Then, we present more theoretical and experimental results of the tilted CF arrays with different $R_{s}$.

First, we study the tilted CF array with $\varepsilon_{d}\neq\varepsilon_{b}$ based on the effective TAM ($\varepsilon_{x'\text{,eff}}=\varepsilon_{d}$,$\varepsilon_{y'\text{,eff}}=\varepsilon_{d}+i\gamma$ with $\gamma=\left(Z_{0}/R_{s}\right)/k_{0}a\cos\alpha$). We know that under $\theta_{i}=-\left(\pi/2-\alpha\right)$ with $\alpha=\arctan\left(\sqrt{\varepsilon_{b}}/\sqrt{\varepsilon_{d}}\right)$, we have $\gamma$-independent PIM and $\gamma$-controlled absorption of transmission waves in the TAM. **Generally, $\gamma$ cannot be too large or too small. Otherwise, the CFs tend to be PEC or air, thus there will be no absorption. Clearly, there exists an optimal value of $\gamma$ to obtain the largest absorption.** To study the optimal value, we derive the relation between $\mathrm{Im}\left(k_{y}\right)$ (i.e. the imaginary part of $k_{y}$) and $\gamma$ under $\theta_{i}=-\left(\pi/2-\alpha\right)$ as,

$\begin{matrix} \left[\mathrm{Im}\left(k_{y}\right)/k_{0}\right]^{3}\left[\gamma^{2}\text{+}\left(1+\varepsilon_{d}\right)^{2}\right]^{2}+4\left[\mathrm{Im}\left(k_{y}\right)/k_{0}\right]^{2}\gamma\sqrt{1+\varepsilon_{d}}\left[\gamma^{2}\text{+}\left(1+\varepsilon_{d}\right)^{2}\right] \\ +\left[\mathrm{Im}\left(k_{y}\right)/k_{0}\right]\left(1+\varepsilon_{d}\right)\left[\gamma^{4}\text{+}\varepsilon_{d}^{2}\left(1+\varepsilon_{d}\right)^{2}+\gamma^{2}\left(5+2\varepsilon_{d}+2\varepsilon_{d}^{2}\right)\right]+2\gamma\left(1+\varepsilon_{d}\right)^{3/2}\left(\gamma^{2}\text{+}\varepsilon_{d}^{2}\right)=0 \end{matrix}$ (S19)

According to Eq. (S19), the relation between $\mathrm{Im}\left(k_{y}\right)$ and sheet resistance $R_{s}$ of the tilted CF array ($a$=5 mm, $d$=30 mm) at 10 GHz is plotted in Fig. S13(a). The red dots and black lines are related to CF arrays with different $\varepsilon_{d}$ and the effective media, respectively. It is seen that **as the increase of $\varepsilon_{d}$, both the optimal sheet resistance and the corresponding maximal value of $\left|\mathrm{Im}\left(k_{y}\right)\right|$ decrease**. Moreover, we notice that **for the experimental sample consisting of PMMA ($\varepsilon_{d}=2.6$) and ITO films (i.e. the sample in Fig. 3 in the Main Text), the optimal sheet resistance is ~$0.35Z_{0}$=132$\boldsymbol{\Omega}$ for the CF array (or ~$0.31Z_{0}$=117$\boldsymbol{\Omega}$ for the effective medium model)**. In experiments, it is not easy to fabricate the ITO films with the exact optimal sheet resistance. Actually, the ITO films we used possess a sheet resistance around 370$\Omega$. In order to make the sheet resistance close to the optimal value, we have stacked two ITO films together, so that the superimposed ITO film has a half sheet resistance ~185$\Omega$, as marked by the blue dashed line in Fig. S13(a). Figure S13(b) shows pictures of the fabricated sample and the ITO films.

**
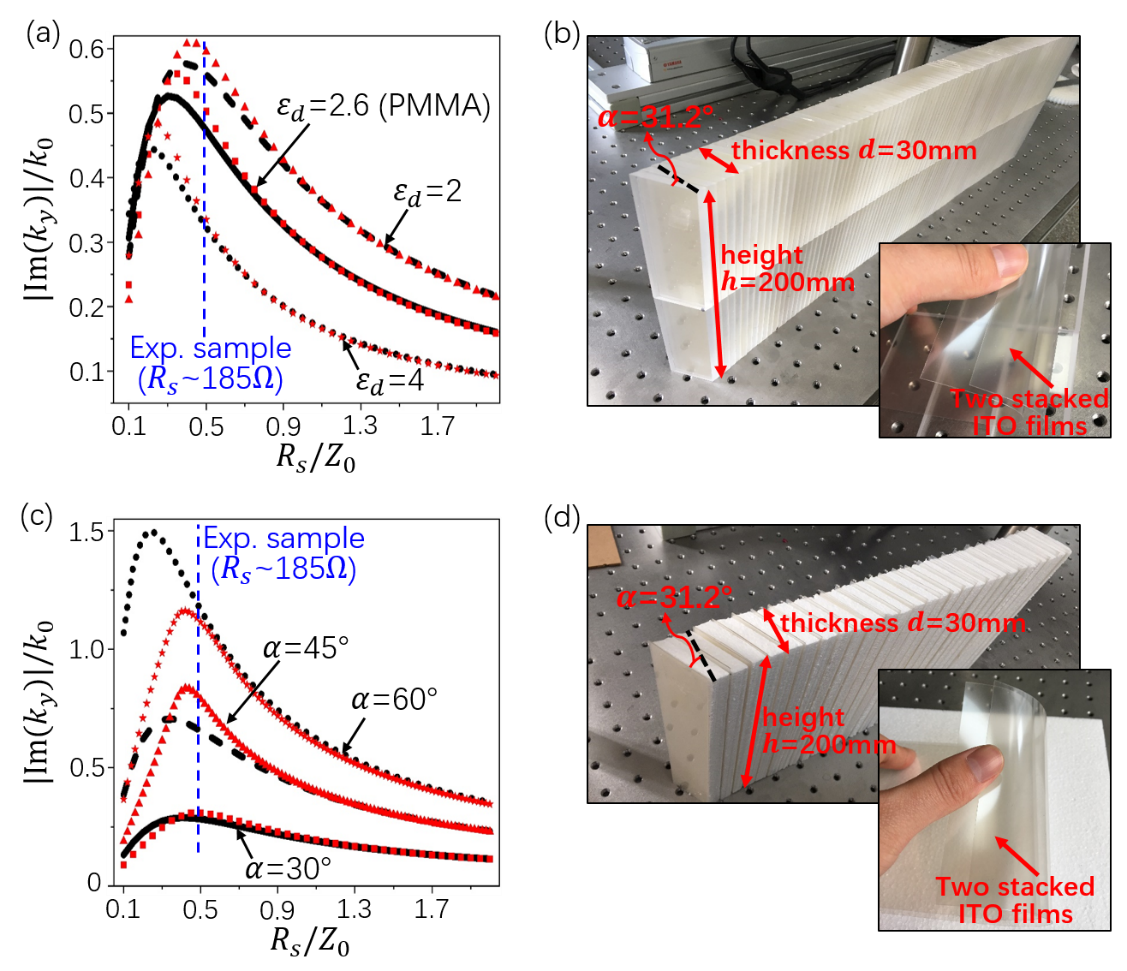
**

**Figure S13. Optimal sheet resistance analysis and details of experimental samples.** [(a) and (c)] $\mathrm{Im}\left(k_{y}\right)$ as the function of sheet resistance $R_{s}$ of tilted CF arrays with (a) $\varepsilon_{d}\neq\varepsilon_{b}$, (c) $\varepsilon_{d}=\varepsilon_{b}$. In (a), the red dots and black lines are related to CF arrays with different $\varepsilon_{d}$ and the effective media, respectively. In (c), the red dots and black lines are related to CF arrays ($\varepsilon_{d}=1$) with different $\alpha$ and the effective media, respectively. [(b) and (d)] Pictures of the fabricated samples and the used ITO films. Two ITO films are stacked together, so that the superimposed film has a sheet resistance around 185$\Omega$. In (b), the sample is composed of PMMA and ITO films (the sample in Fig. 3). In (d), the sample is composed of foam and ITO films (the sample in Fig. 4).

Second, we study the tilted CF array with $\varepsilon_{d}=\varepsilon_{b}=1$. Based on complex dispersion of the effective medium model, we obtain the $\mathrm{Im}\left(k_{y}\right)$ under $\theta_{i}=-\alpha$ as,

$\mathrm{Im}\left(k_{y}\right)/k_{0}=\frac{8\gamma\sin\alpha\sin2\alpha}{8+3\gamma^{2}+\gamma^{2}\left(4\cos2\alpha+\cos4\alpha\right)}$ (S20)

From Eq. (S20), we can find out the maximal $\left|\mathrm{Im}\left(k_{y}\right)\right|$ as

$\left[\left|\mathrm{Im}\left(k_{y}\right)\right|/k_{0}\right]_{\max}=\sin\alpha\tan\alpha$ (S21)

at the optimal $\gamma$ or sheet resistance

$\gamma=1+\tan^{2}\alpha$ or $R_{s}=\frac{\cos\alpha}{k_{0}a}Z_{0}$ (S22)

From Eq. (S22), we find out **the optimal sheet resistance of the experimental sample consisting of foam and ITO films (the sample in Fig. 4 in the Main Text) as ~$0.41Z_{0}$=155**$\boldsymbol{\Omega}$, as shown in Fig. S13(c). Similarly, we have stacked two ITO films together to construct a superimposed ITO film with $R_{s}$~185$\Omega$, as shown by pictures of the experimental sample and ITO films in Fig. S13(d). In addition, Eqs. (S20)-(S22) indicate an interesting thing, that is, **the maximal $\left|\mathrm{Im}\left(k_{y}\right)\right|$ increases as the increase of $\alpha$, and tends to infinity as $\alpha$ goes to $90^{\circ}$, as shown in Fig. S13(c). This means that perfect absorption of electromagnetic/optical waves can be realized by using ultrathin tilted CF arrays with $\alpha\to90^{\circ}$ in an ultra-broad frequency band**.

**
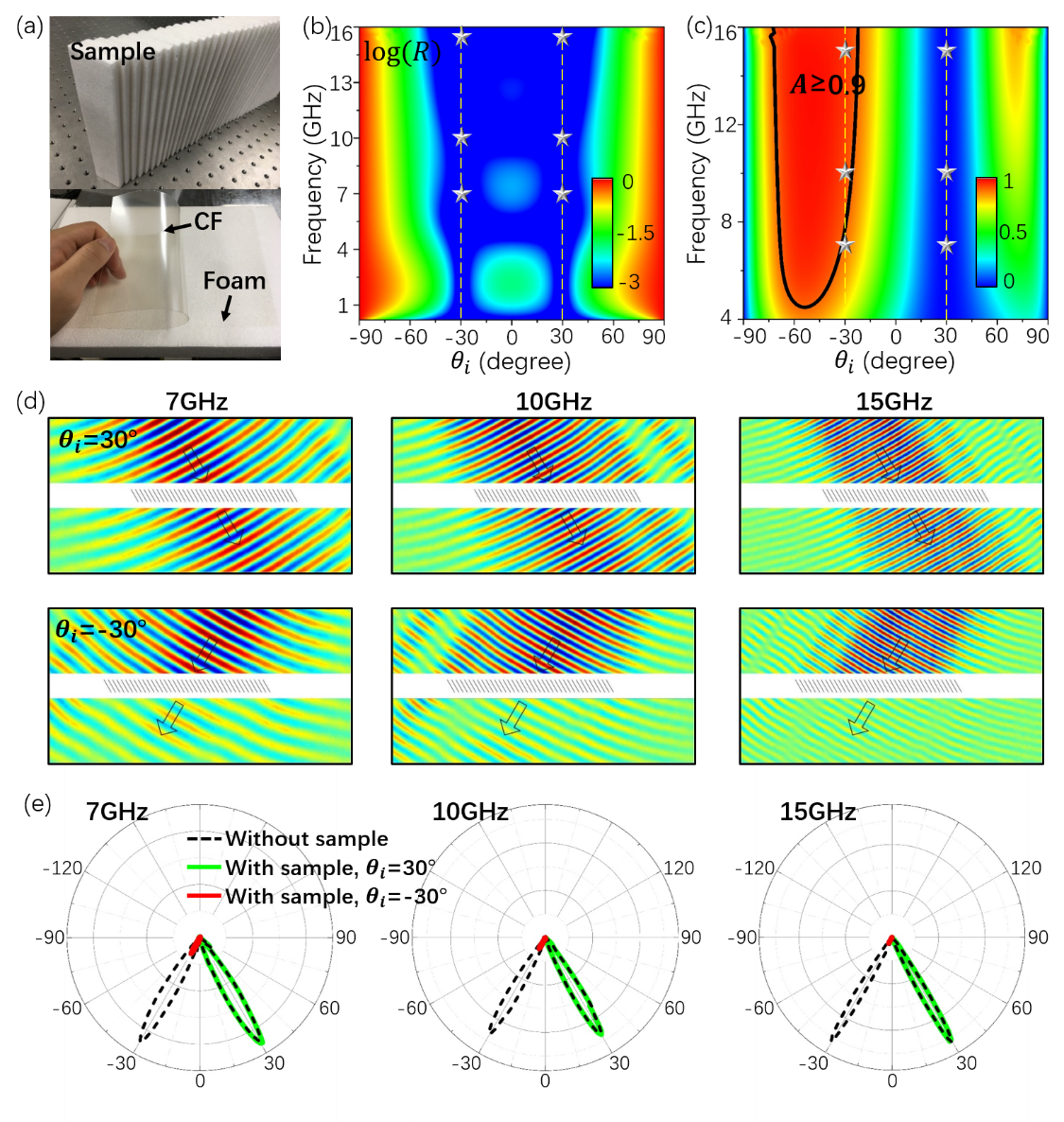
**

**Figure S14.** **Experimental demonstration of ultra-broadband PIM and near-perfect absorption by the tilted CF array (foam and ITO films) with $\alpha=30^{\circ}$, $a$=10 mm and $d$=30 mm. There is only one ITO film (**~370Ω**) between two adjacent foam layers.** (a) Photograph of the fabricated sample. Calculated (b) reflectance, (c) absorptance $A$ of the sample as functions of the $\theta_{i}$ and working frequency. The stars denote the cases verified in experiments. (d) Measured near-field electric fields under $\theta_{i}=30^{\circ}$ (upper) and $\theta_{i}=-30^{\circ}$ (lower) at 7 GHz (left), 10 GHz (middle) and 15 GHz (right). (e) Measured far-field radiation power in the absence of sample (black dashed lines), with sample under $\theta_{i}=30^{\circ}$ (green lines) and $\theta_{i}=-30^{\circ}$ (red lines) at 7 GHz (left), 10 GHz (middle) and 15 GHz (right).


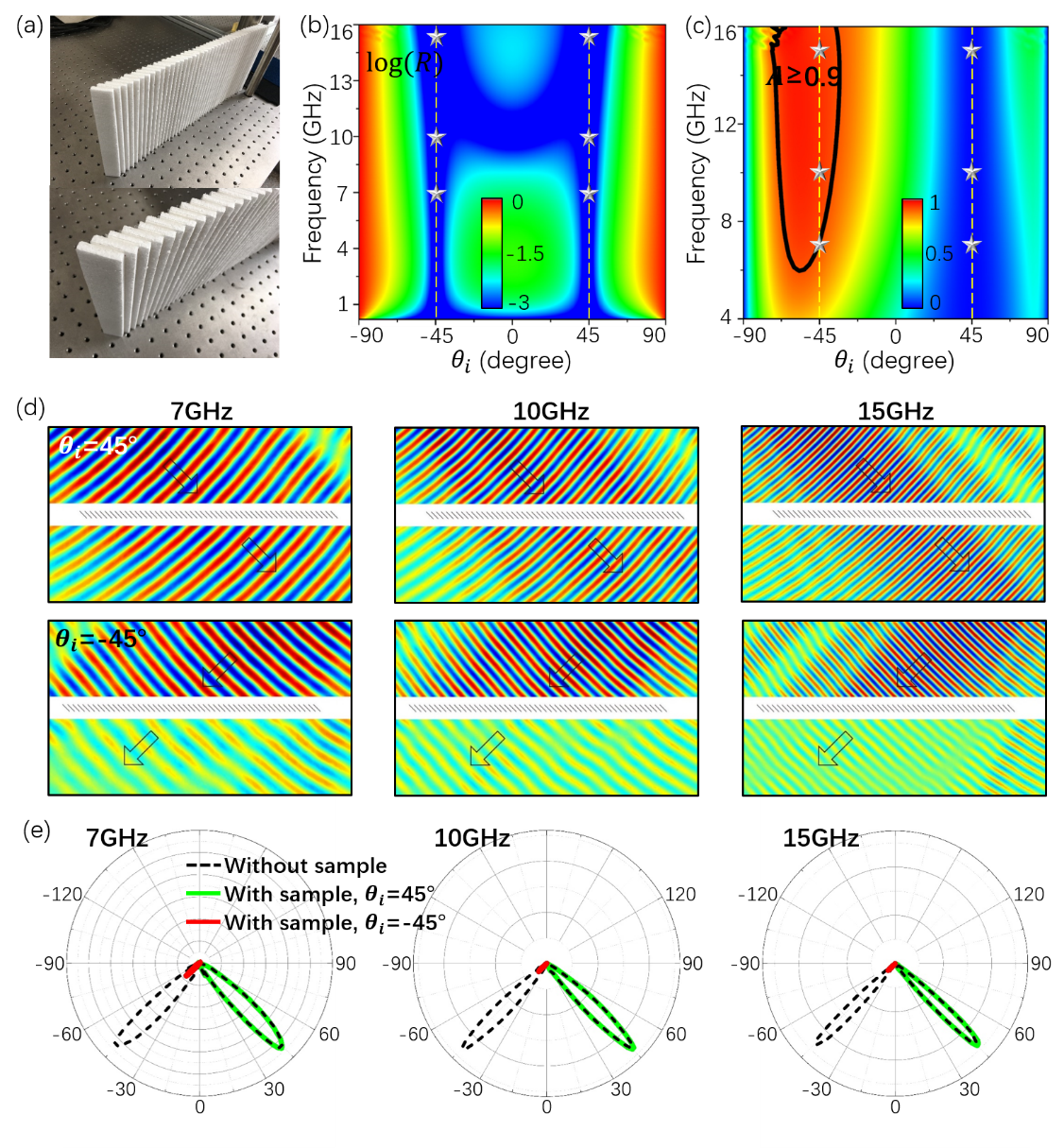


**Figure S15.** **Experimental demonstration of ultra-broadband PIM and near-perfect absorption by the tilted CF array (foam and ITO films) with $\alpha=45^{\circ}$, $a$=10 mm and $d$=15 mm. There is only one ITO film (**~370Ω**) between two adjacent foam layers.** (a) Photograph of the fabricated sample. Calculated (b) reflectance, (c) absorptance $A$ of the sample as functions of the $\theta_{i}$ and working frequency. The stars denote the cases verified in experiments. (d) Measured near-field electric fields under $\theta_{i}=45^{\circ}$ (upper) and $\theta_{i}=-45^{\circ}$ (lower) at 7 GHz (left), 10 GHz (middle) and 15 GHz (right). (e) Measured far-field radiation power in the absence of sample (black dashed lines), with sample under $\theta_{i}=45^{\circ}$ (green lines) and $\theta_{i}=-45^{\circ}$ (red lines) at 7 GHz (left), 10 GHz (middle) and 15 GHz (right).

Next, we have performed more numerical calculations and microwave experiments to show the influences of sheet resistance on the absorption performance. Figure S14 discusses the sample consisting of alternative foam and ITO films ($a$=10 mm, $\alpha=30^{\circ}$), whose thickness and height are 30 mm and 200 mm, respectively. Different from the sample in Fig. 4 in the Main Text, here there is only one ITO film between two adjacent foam layers, as shown by pictures of the fabricated sample and ITO film in Fig. S14(a). Figures S14(b) and S14(c), respectively, present the simulated reflectance and absorptance on the fabricated sample, showing the zero reflection for all frequencies <16 GHz under $\theta_{i}=\pm30^{\circ}$ and near-perfect absorption under $\theta_{i}=-30^{\circ}$. These results have been confirmed by experimental measured electric fields under $\theta_{i}=30^{\circ}$ (upper) and $\theta_{i}=-30^{\circ}$ (lower) at 7 GHz (left), 10 GHz (middle) and 15 GHz (right) in Fig. S14(d). We have also measured the far-field radiation patterns (green and red lines) in Fig. S14(e). The black dashed lines denote the reference patterns in the absence of the sample. **These results show that the absorption is decreased a bit in comparison with the results in Fig. 4 in the Main Text, because the sheet resistance here is away from the optimal value (i.e. ~155$\boldsymbol{\Omega}$). Even so, ultra-broadband PIM and quite good absorption performance can still be observed, manifesting the robustness of wave absorption by the tilted CF arrays.**

Then, we change the rotation angle to $\alpha=45^{\circ}$, and reduce the thickness to 15 mm. Figure S15(a) shows the fabricated sample. Figures S15(b) and S15(c), respectively, present the simulated reflectance and absorptance on the fabricated sample, showing the zero reflection for all frequencies <16 GHz under $\theta_{i}=\pm45^{\circ}$ and near-perfect absorption under $\theta_{i}=-45^{\circ}$. These results have been confirmed by experimental measured electric fields under $\theta_{i}=45^{\circ}$ (upper) and $\theta_{i}=-45^{\circ}$ (lower) at 7 GHz (left), 10 GHz (middle) and 15 GHz (right) in Fig. S15(d). We have also measured the far-field radiation patterns (green or red lines) in Fig. S15(e). The black dashed lines denote the reference patterns in the absence of the sample. **These results show that although the thickness is halved here, the absorption performance is even better than that in Fig. S15 with $\alpha=30^{\circ}$. Actually, as the rotation angle increases, ultra-broadband perfect absorption of electromagnetic waves can be realized even in deep-subwavelength CF arrays**.

## Experimental setup and measurement methods

In experiments, we measure the near-field electric fields with the experimental setup shown in Fig. S16(a). An emitting horn antenna is placed ~0.8 meter away from the sample to generate the incident waves. A probing antenna is used to probe the near-field electric fields before and after the sample. The scanning rectangular area is of 700×150 mm^2^ (or 600×150 mm^2^) before and after the sample consisting of PMMA (or foam) and ITO films. The scanning areas are on the central plane of the sample. The probing antenna is mounted to a computer controlled translational stage (not shown here). Both the probing antenna and the emitting horn antenna are connected to a network analyzer (KEYSIGHT PNA Network Analyzer N5224B) for data acquisition.


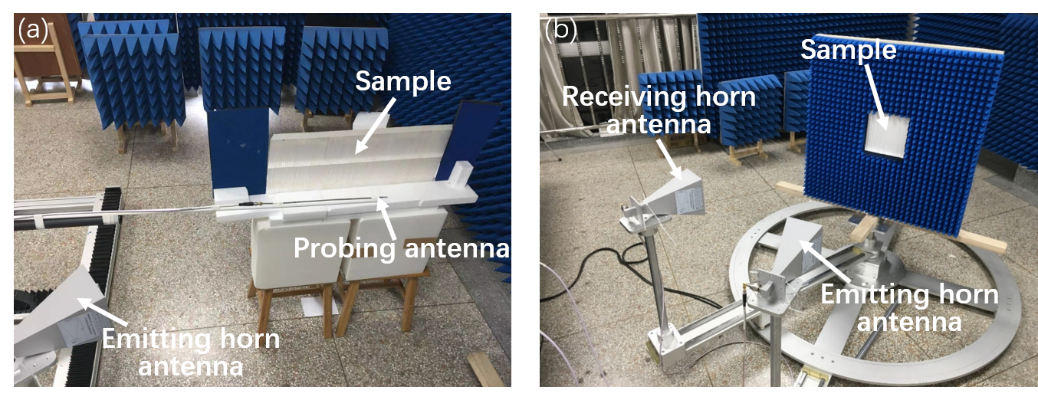


**Figure S16. Experimental setup and measurement methods.** Pictures of experimental setup for the measurement of (a) near-field electric fields, (b) far-field power radiation patterns.

We note that only the electric fields perpendicular to the propagation direction are measured because of the selectivity of the probing antenna in measurement. Therefore, in the scan area before the sample, the measured electric fields come from the incident waves and a part of the possible reflection waves except of the special case of $\theta_{i}=\pm45^{\circ}$. **In order to further confirm the zero reflection and near-perfect absorption in experiments, we have also measured the electric fields in the absence of samples and the far-field power radiation patterns (see experimental setup in Fig. S16(b))**. Regarding to the experiments in Fig. 3 in the Main Text, we have also measured near-field electric fields in the absence of samples under $\theta_{i}=58.2^{\circ}$ at 7 GHz (left), 10 GHz (middle) and 15 GHz (right), as shown in Fig. S17. Compared with the field distributions with samples in Fig. 3, we can that the electric fields before the sample are almost the same. This demonstrates that there are almost no reflection waves in the existence of samples (Fig. 3), thus confirming the ultra-broadband PIM and near-perfect absorption. Regarding to the experiments in Fig. 4 in the Main Text, we have measured far-field power radiation patterns under $\theta_{i}=30^{\circ}$ (green lines) and $\theta_{i}=-30^{\circ}$ (red lines) at 7 GHz (left), 10 GHz (middle) and 15 GHz (right), as shown in Fig. S18. The black dashed lines denote the reference patterns in the absence of the sample. These results clearly demonstrate the ultra-broadband PIM and near-perfect absorption.


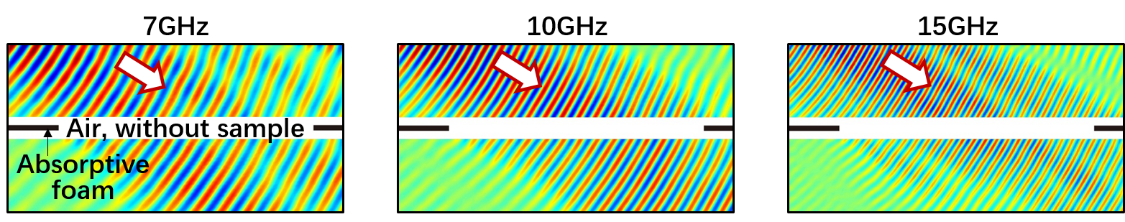


**Figure S17. Measured electric fields in the absence of samples for a reference (regarding to the sample in Fig. 3).** Measured near-field electric fields in the absence of samples under the incident angle of $\theta_{i}=58.2^{\circ}$ at 7 GHz (left), 10 GHz (middle) and 15 GHz (right)

**
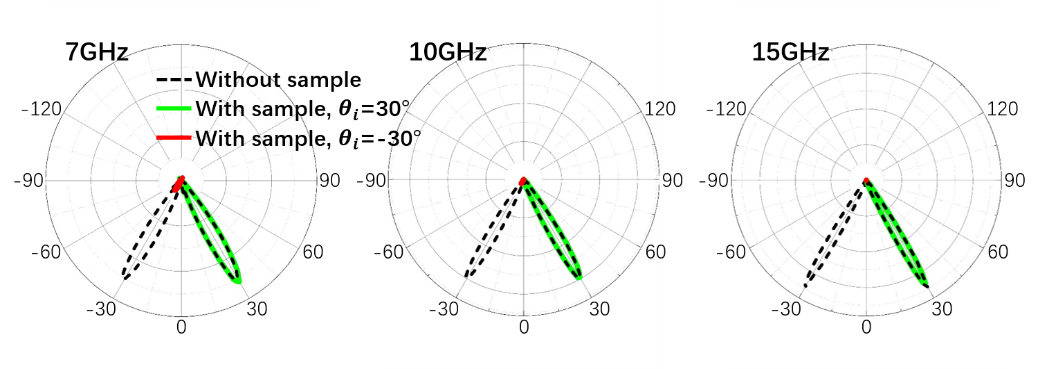
**

**Figure S18. Measured far-field power radiation patterns (regarding to the sample in Fig. 4).** Measured far-field power radiation patterns for the sample consisting of foam and ITO films under $\theta_{i}=30^{\circ}$ (green lines) and $\theta_{i}=-30^{\circ}$ (red lines) at 7 GHz (left), 10 GHz (middle) and 15 GHz (right). The black dashed lines denote the reference patterns in the absence of samples.

In the far-field experiments, the power radiation pattern of the sample is measured with the experimental setup shown in Fig. S17(b). An emitting horn antenna is placed 1m away from the sample to generate the incident waves. A receiving horn antenna placed at the same distance is used to measure the radiation pattern. The receiving horn antenna can be freely moved around the sample so that we could receive scattering signals in all directions. Both the emitting and receiving horn antennas are connected to a vector network analyzer (KEYSIGHT PNA Network Analyzer N5224B) for data acquisition. The power radiation pattern in the absence of sample is measured as a reference.


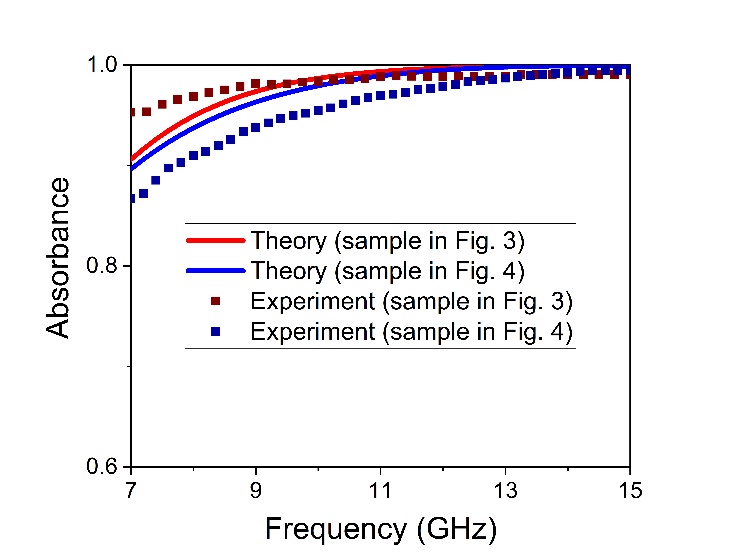


**Figure R19**. **Absorptance of the experimental samples within the measuring frequency range 7-15 GHz.** The red and blue lines/dots are related to the sample in Fig. 3 (incident angle -30°) and the sample in Fig. 4 (incident angle -58.2°), respectively. The lines and dots denote the theoretical and experimental results, respectively.

Furthermore, through integrating far-field power for all directions, the absorbance by the designed absorbers can be evaluated. Figure S19 presents the absorptance distributions of the experimental sample in Fig. 3 (incident angle -30$^{\circ}$) and the experimental sample in Fig. 4 (incident angle -58.2$^{\circ}$), as shown by the red and blue lines, respectively. The experimental results (dots) are in good coincidence with the theoretical results (lines). The absorption is quite high within the measuring frequency range 7-15 GHz. The relatively low absorption at low frequencies attributes to the long wavelengths. Through increasing the sample thickness, the absorption performance can be greatly improved.

## Improvement of absorption by using reflectors

In the Main Text, the absorption by the tilted CF array is asymmetric with respect to $\theta_{i}$, and is small under $\theta_{i}>0$. Here, we show that the absorption can be greatly improved by using a reflector. Figure S20 (a) presents the absorptance by the sample in Fig. 3 in the Main Text, but with a PEC reflector behind. Interestingly, symmetric high absorption (>0.9) in an ultra-broad frequency band (4~16 GHz) under $\theta_{i}=\pm58.2^{\circ}$ is seen, which is further confirmed by the simulations in Fig. S20(b). The similar symmetric high absorption is also observed by the sample in Fig. 4 in the Main Text with a PEC reflector behind, as shown in Figs. S20(c) and S20(d). **The results clearly show that with a PEC reflector, symmetric absorption can be obtained, and both the angular performance and band width of absorption can be greatly improved.**


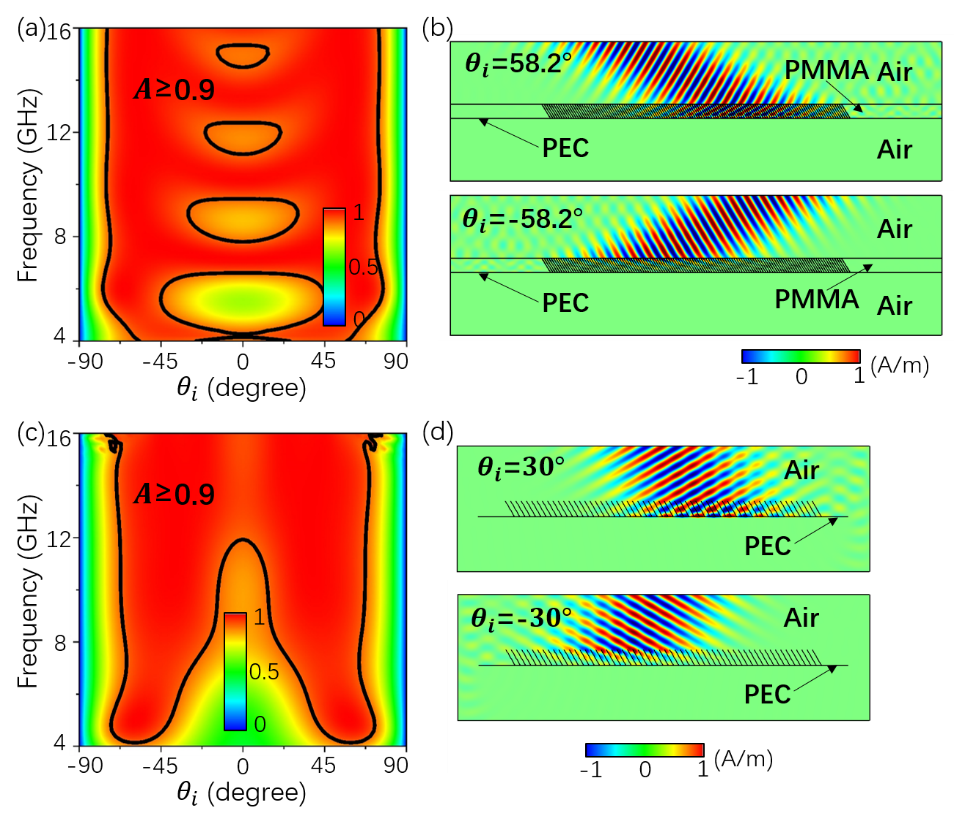


**Figure S20. Improving absorption by using PEC reflectors.** (a) Absorptance by the experimental sample (PMMA and ITO films) with a PEC reflector as functions of $\theta_{i}$ and working frequency. (b) Simulated magnetic field-distributions under $\theta_{i}=\pm58.2^{\circ}$ at 10 GHz. The sample in (a) and (b) is the same as that in Fig. 3. (c) Absorptance by the experimental sample (foam and ITO films) with a PEC reflector as functions of $\theta_{i}$ and working frequency. (b) Simulated magnetic field-distributions under $\theta_{i}=\pm30^{\circ}$ at 10 GHz. The sample in (c) and (d) is the same as that in Fig. 4.

**References**

1 Feng, S. & Halterman, K. Coherent perfect absorption in epsilon-near-zero metamaterials. *Physical Review B* **86**, 165103 (2012).

2 Luo, J., Li, S., Hou, B. & Lai, Y. Unified theory for perfect absorption in ultrathin absorptive films with constant tangential electric or magnetic fields. *Physical Review B* **90**, 165128 (2014).

3 Nefedov, I. S., Valagiannopoulos, C. A., Hashemi, S. M. & Nefedov, E. I. Total absorption in asymmetric hyperbolic media. *Scientific Reports* **3**, 2662 (2013).

4 Maxwell Garnett, J. C. Colours in metal glasses and in metallic films. *Philosophical Transactions of the Royal Society A: Mathematical, Physical and Engineering Sciences* **203**, 385-420 (1904).
